# MBNL proteins in health, disease, and therapeutic applications

**DOI:** 10.1093/nar/gkag262

**Published:** 2026-04-02

**Authors:** Nikola Musiała-Kierklo, Patryk Konieczny, Patrycja Plewka, Adam Jasiok, Ewa Stępniak-Konieczna

**Affiliations:** Laboratory of RNA Biology, Department of Biochemistry and Biotechnology, Poznan University of Life Sciences, Dojazd 11, 60-632 Poznan, Poland; Doctoral School of Natural Sciences, Adam Mickiewicz University, Uniwersytetu Poznanskiego 2, 61-614 Poznan, Poland; Institute of Human Biology and Evolution, Adam Mickiewicz University, Uniwersytetu Poznanskiego 6, 61-614 Poznan, Poland; Laboratory of RNA Biology, Department of Biochemistry and Biotechnology, Poznan University of Life Sciences, Dojazd 11, 60-632 Poznan, Poland; Laboratory of RNA Biology, Department of Biochemistry and Biotechnology, Poznan University of Life Sciences, Dojazd 11, 60-632 Poznan, Poland; Poznan University of Life Sciences Doctoral School, Collegium Maximum, Wojska Polskiego 28, 60-637 Poznan, Poland; Laboratory of RNA Biology, Department of Biochemistry and Biotechnology, Poznan University of Life Sciences, Dojazd 11, 60-632 Poznan, Poland

## Abstract

The Muscleblind-like (MBNL) family comprises evolutionarily conserved RNA-binding proteins that interact with target RNAs via zinc finger domains. MBNLs orchestrate RNA processing, particularly alternative splicing, driving the developmental fetal-to-adult isoform switch across numerous target transcripts. This transition is a cornerstone in the process of MBNL-maintained cellular homeostasis and fails in many pathological conditions associated with deregulated expression or function of specific MBNL paralogs. This review provides current insights into the roles of *MBNL* genes and proteins in both health and disease. We examine their genomic architecture and protein organization and synthesize key insights from animal models to delineate the selective and compensatory functions of individual MBNL paralogs in physiology. To illustrate the roles of MBNLs in disease, we outline nucleotide repeat expansion disorders marked by their functional depletion, with a primary focus on myotonic dystrophy (DM). We also highlight selected cancer studies that have demonstrated the dual roles of MBNLs in tumorigenesis, encompassing both pro-oncogenic and tumor suppressive functions. Finally, using DM as a model, we review evidence for the therapeutic potential of endogenous *MBNL* gene modulation and argue that analogous strategies could be adapted and tailored to restore MBNL homeostasis in other disorders involving their dysregulation.

## Graphical Abstract

**Graphical Abstract:**
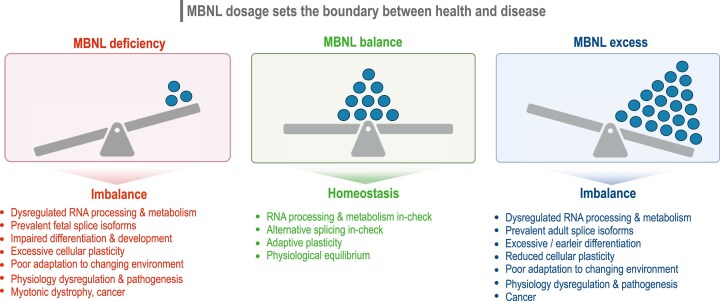

## Introduction

The Muscleblind-like (MBNL) family comprises highly conserved RNA-binding proteins (RBPs) which interact with target RNAs via zinc finger (ZnF) domains. Mammalian MBNLs include three paralogs (MBNL1, 2, and 3) which, in essence, function as master regulators of cellular RNA metabolism and homeostasis. Among their multifaceted roles in RNA processing—including the regulation of alternative polyadenylation, RNA stability and localization, microRNA biogenesis, and circular RNA generation—one of their most prominent functions involves the regulation of alternative splicing (AS) [1].

MBNL proteins share conserved structural features but differ largely in functional specializations and developmental expression patterns. *MBNL1* and *MBNL2* are expressed at low levels in fetal and undifferentiated cells but increase markedly during differentiation, showing widespread expression across diverse adult tissues, with the highest abundance in muscle [2–4]. Although both are largely coexpressed and functionally compensatory, MBNL1 is the predominant paralog, critical for AS transitions in muscle differentiation [4, 5], whereas MBNL2 is enriched in the brain, where it governs key developmental splicing programs [6]. Together, MBNL1 and MBNL2 act as developmental regulators that repress embryonic splice patterns in favor of adult splice isoforms. In contrast, MBNL3 is expressed primarily during early development, especially in the placenta, and only transiently in regenerating adult muscle [7]. Unlike MBNL1 and MBNL2, MBNL3-regulated AS suppresses differentiation-associated transcripts and appears to function oppositely to the other two paralogs by inhibiting, rather than promoting, muscle differentiation [8]. Failure of MBNL-regulated RNA processing occurs in multiple pathologies, arising either from MBNL sequestration and functional insufficiency or from dysregulated *MBNL* gene expression.

Here, we explore the roles of MBNLs in health and disease, with particular emphasis on their crucial functions in AS. We synopsize the organizational and structural characteristics of *MBNL* genes and proteins, highlighting their common and unique features. Insights from animal models of *MBNL* genes deficiency are integrated to illustrate the principal tissue-specific functions of individual paralogs and to delineate their selective and compensatory roles. The pathophysiological relevance of MBNLs is discussed in the context of myotonic dystrophy (DM), the most extensively characterized disorder marked by their functional deficiency, as well as other nucleotide repeat expansion diseases involving MBNL depletion. We also illustrate the basic mechanisms through which MBNLs affect cancer hallmarks and thus may play dual, tumor-suppressive or pro-oncogenic roles. Finally, using DM as a model, we discuss endogenous modulation as a promising strategy to restore MBNL balance. Collectively, the available evidence supports MBNLs as promising therapeutic targets, with emerging precision gene-tuning strategies potentially applicable to other disorders involving their dysregulation.

## *MBNL* genes—structure, regulation, and splicing

*MBNL* genes share a high degree of functional and structural conservation across multicellular organisms. Human orthologs of *MBNL* genes exist in diverse species including *Drosophila melanogaster* [9, 10], *Caenorhabditis elegans* [11], and *Danio rerio* [12, 13], as well as higher vertebrates and mammals, including *Mus musculus* [14]. Historically, Muscleblind (Mbl) was identified as a protein essential for photoreceptor and muscle differentiation in *Drosophila melanogaster* [9, 10], whereas the mammalian homolog was first cloned as a polypeptide that binds the CUG repeat expanded RNA underlying the genetic multiorgan disorder myotonic dystrophy [3, 15–17].

Subsequent studies identified three distinct homologous mammalian genes, including *MBNL1* (formerly *MBNL/EXP*), *MBNL2* (formerly *MBLL/MPL1*), and *MBNL3* (formerly *MBXL/CHCR*), mapped to chromosomes 3, 13, and X, respectively [3, 4, 18], and demonstrated their role as AS regulators [19, 20]. In contrast to invertebrates, which only have a single *Mbl* gene, the three homologs present in most vertebrate species likely arose as a consequence of gene duplication of a common ancestor [21].

### *MBNL* promoters

**Figure 1.**
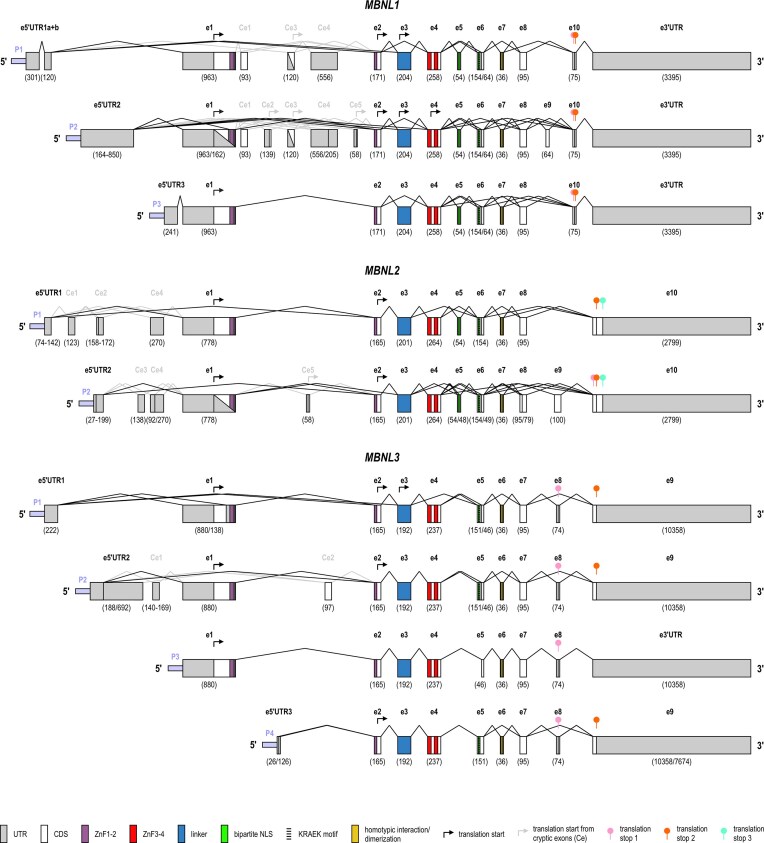
Genomic organization of human *MBNL* genes and promoters. Schematics (not to scale) showing the genomic organization of the *MBNL1, MBNL2*, and *MBNL3* genes and paralog-dependent splicing of exons in transcripts derived from alternative promoters. Coding exons (e) and cryptic exons (Ce) are marked, and numbers in parentheses indicate nucleotide lengths. The numbers in parentheses separated by a slash indicate alternative shorter and longer versions of that exon, whereas numbers separated by a hyphen denote the range of exon lengths. The features are described in the legend. The diagram is based on the RefSeq *MBNL1–3* sequences available in the UCSC Genome Browser (GRCh38/hg38) and NCBI Gene.

A candidate approach in *Drosophila Mbl* identified two putative promoter regions, P1 and P2, involving the beginnings of exons 1 and 2, respectively, that were able to initiate transcription [22]. Similarly, tissue-specific expression of *MBNL* genes is determined by alternative use of *MBNL* promoters and 5′ untranslated (UTR) initiation exons (e5′UTRs) comprising important regulatory elements [22–24] (Fig. 1). In human *MBNL1*, these include three promoters (P1–P3) and corresponding transcription initiation sites associated with three alternative e5′UTRs [2, 21, 24]. Among these, P2 showed the highest activity in most tissues including the fetal and adult heart and skeletal muscle, whereas P3 was found to be predominant in the brain, fetal liver, spleen, and thymus [24]. P1 generally displays marginal activity compared with P2 and P3; however, its significant activity was detected in the liver, fetal spleen and thymus [24]. This tissue-specific complexity of gene expression is also evident for the other two *MBNL* genes, with two promoters annotated for *MBNL2* and four promoters for *MBNL3*. Importantly, they not only regulate the tissue-specific gene expression but also determine the function of the ensuing proteins.

For example, AS of the first coding exon (e1) of *MBNL1*, which is critical for protein turnover and splicing activity, was reported in transcripts derived from P2 [24]. Another example is a truncated MBNL3 protein isoform with a single ZnF pair (ZnF3–4), generated via the use of a novel eutherian-specific promoter (marked as P4 in Fig. 1), which occurs specifically in placental tissue [25].

### Regulation of *MBNL* expression

Little is known about the transcription factors (TFs) that contribute to promoter-differential expression of *Mbl* and *MBNL* genes. Multiple *cis-*regulatory modules around *Mbl* promoters have been shown to bind Myocyte enhancer factor 2 (Mef2) as well as the muscle organizing factors Biniou (Bin), Tinman (Tin), and Twist [22]. A crucial muscle enhancer (ME) of *Mbl* expression in the embryonic somatic musculature was found downstream of P2. Moreover, sequences located farther downstream, harboring additional binding sites for TFs essential for central nervous system (CNS) development, were identified as the neural-specific *Mbl* enhancer (NE).

Importantly, the same study revealed functionally conserved human *MBNL1* promoter regions, Hsa-P1 and Hsa-P2 [22], which overlap human P2 and P3, respectively [24]. Notably, *Drosophila* ME drove the expression of a reporter gene in the embryonic musculature only when fused to Hsa-P1 but not Hsa-P2, indicating the relevance of this genomic region in muscle-specific *MBNL1* expression [22].

Murine *Mbnl1* harbors important enhancer activity within methylated region 2 (MeR2), which is located in the intron preceding the first coding exon [26], and corresponds to P3 preceding the first coding exon in human *MBNL1*. While MeR2 is methylated during myogenic differentiation, suppression of methylation dramatically increases RNAPII occupancy around this region and enhances *Mbnl1* transcription starting at the upstream exon, suggesting that MeR2 has enhancer activity [26]. Considering that similar enhancer regions in intron 1 have been identified in several muscle-specific genes, e.g. *Dmd* [27] and *Ache* [28], MeR2, as well as the corresponding P3 region in humans, may be critical for muscle-specific *MBNL* expression [26].

Substantial evidence indicates that the 3′UTR exons of all *MBNL* paralogs contain binding sites for multiple miRNA regulators of *MBNL* transcripts [29, 30]. For example, the miR-30-5p family, which suppresses muscle differentiation, directly binds 3′UTR sequences of all *MBNL* paralogs to inhibit their expression [29]. Furthermore, negative regulation of *MBNL1* and *MBNL2* transcripts by miR-23b and miR-218 has recently been exploited in an experimental therapy against DM, in which antisense oligomers were designed to prevent miRNA-mediated translational repression and increase protein availability [30–33].

### *MBNL* coding sequence

**Figure 2.**
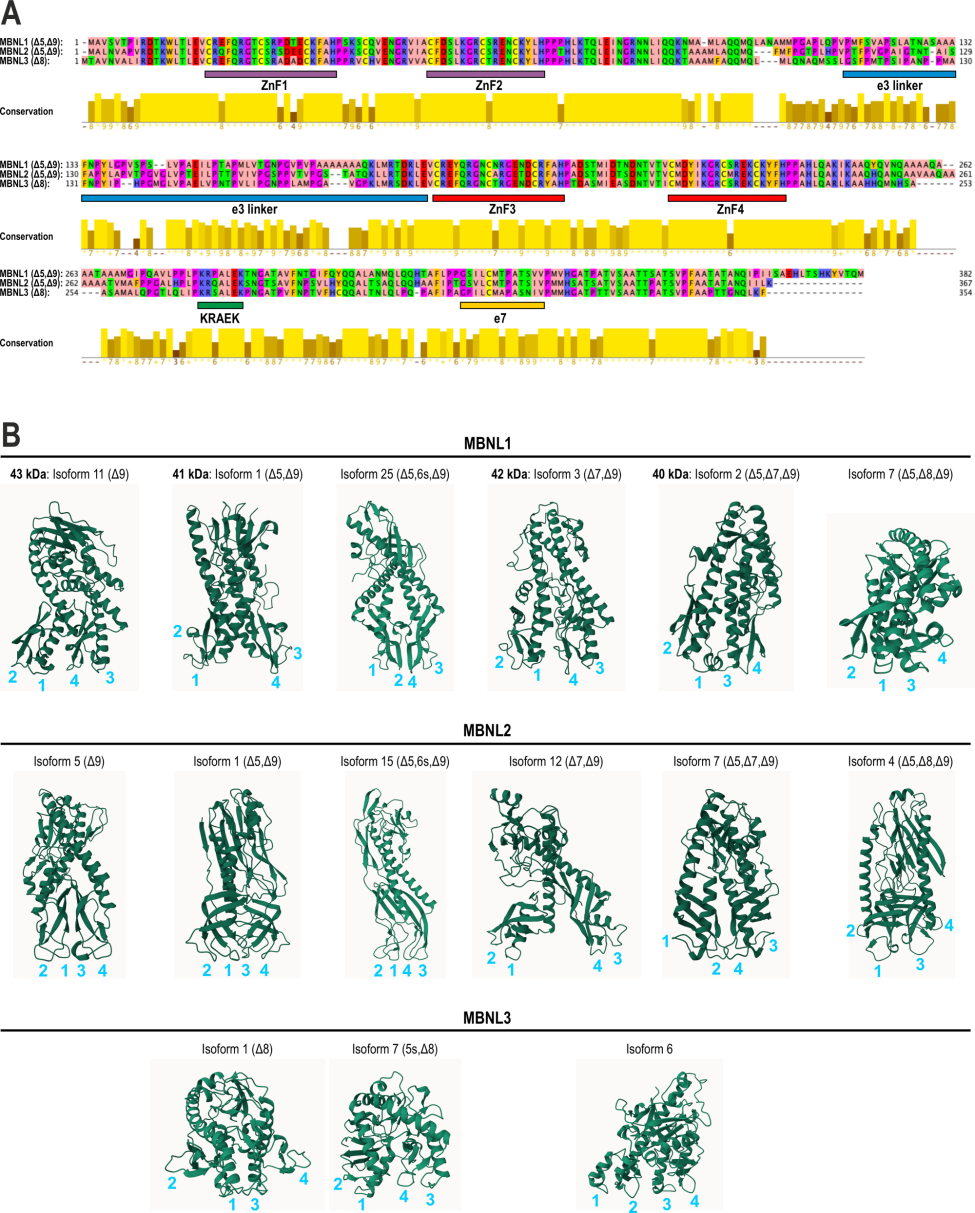
Comparison of major protein isoforms encoded by *MBNL* paralogs. (**A**) Comparison of the amino acid sequences of the MBNL1, MBNL2, and MBNL3 isoforms, which lack the same exons, with distinct features marked, including the ZnFs, the KRAEK motif, the e3 linker, and the homodimerization determinant e7. Note that MBNL3 lacks the e5 coding sequence, and its e8 corresponds to e9 of MBNL1–2. The MBNL1–3 RefSeq sequences were obtained from the NCBI Gene and aligned via Jalview software (version 2.11.5.1). The isoforms selected for sequence alignment in (A) include MBNL1 isoform 1 (Table 1), MBNL2 isoform 1, and MBNL3 isoform 1 (Table 2). (**B**) 3D structures of the major protein isoforms encoded by the indicated isoforms of MBNL1, MBNL2, and MBNL3 with the positions of ZnFs 1–4 indicated. 6s (for MBNL1 and MBNL2) and 5s (for MBNL3) indicate alternative shorter versions of these exons, as marked in Fig. 1. Note that the corresponding isoforms of the MBNL1–3 paralogs are arranged vertically. The 3D structure predictions were obtained via the Boltz-2 model running on the Benchling infrastructure.

All three human *MBNL* genes share structural similarities, and individual exons are subjected to similar AS (Fig. 1, and Tables 1 and 2). *MBNL1* contains two constitutive exons (e4 and e10) and eight alternatively spliced exons (e1–e3 and e5–e9). In *MBNL2* and *MBNL3*, e4 is alternatively spliced, whereas *MBNL3* lacks the corresponding e5 region. At the level of sequence, structure, and function, MBNL3 is also more distinct from the other two paralogs (Figs 1 and 2). The cryptic exons present in all paralogs may be associated with alternative translation initiation sites as indicated in Fig. 1.

**Table 1.** Human MBNL1 protein isoforms

MBNL1							
Isoform	Length (aa)	Mol Mass (Da)	kDa	Promoters	Coding exons	ΔExons (1–9)	Transl. Stop
**1**	**382**	**40 991.96**	**41**	**P1, P2, P3**	**1,2,3,4,6,7,8,10**	**5,9**	**2**
**2**	**370**	**39 830.58**	**40**	**P2, P3**	**1,2,3,4,6,8,10**	**5,7,9**	**2**
**3**	**388**	**41 816.85**	**42**	**P2, P3**	**1,2,3,4,5,6,8,10**	**7,9**	**2**
4	302	33 049.76	33	P2	1,2,4,6,8,10	3,5,7,9	2
5	314	34 211.14	34	P1, P2	1,2,4,6,7,8,10	3,5,9	2
6	340	36 991.46	37	P2	1,2,3,4,8,9,10	5,6,7	2
7	342	37 073.42	37	P1, P2, P3	1,2,3,4,6,7,10	5,8,9	1
8	343	36 550.93	37	P2	Ce5,2,3,4,5,6,7,8,10	1,9	2
9	348	37 898.31	38	P2, P3	1,2,3,4,5,6,10	7,8,9	1
10	431	46 495.26	46	P1, P2	1,Ce1,2,3,4,5,6,7,8,10	9	2
**11**	**400**	**42 978.23**	**43**	**P1, P2, P3**	**1,2,3,4,5,6,7,8,10**	**9**	**2**
12	360	39 059.69	39	P2	1,2,3,4,5,6,7,10	8,9	1
13	330	35 912.04	36	P2, P3	1,2,3,4,6,10	5,7,8,9	1
14	326	34 677.81	35	P1, P2	Ce3,2,3,4,6,7,8,10	1,5,9	2
15	320	35 036.03	35	P2	1,2,4,5,6,8,10	3,7,9	2
16	308	32 437.19	32	P1, P2	2,3,4,5,6,7,8,10	1/1l,9	2
17	290	30 450.92	30	P1, P2	2,3,4,6,7,8,10	1/1l,5,9	2
18	278	29 289.55	29	P1, P2	2,3,4,6,8,10	1,5,7,9	2
19	266	27 928.81	28	P1, P2	3,4,6,7,8,10	1,2,5,9	2
20	243	25 541.22	26	P2	2,3,4,6s,8,10	1,5,7,9	2
21	238	25 371.00	25	P2	2,3,4,6,10	1,5,7,8,9	1
22	219	24 528.06	25	P3	1,2,4,10	3,5,6,7,8,9	2
23	413	44 508.99	45	P1, P2	1,Ce1,2,3,4,6,7,8,10	5,9	2
24	401	43 347.61	43	P2	1,Ce1,2,3,4,6,8,10	5,7,9	2
25	347	37 243.64	37	P1	1,2,3,4,6s,7,8,10	5,9	2
26	344	36 664.08	37	P2	Ce3,2,3,4,5,6,7,8,10	1,9	2
27	332	36 197.41	36	P2, P3	1,2,4,5,6,7,8,10	3,9	2
29	326	34 692.83	35	P2	Ce2,2,3,4,6,7,8,10	1,5,9	2
30	325	34 564.66	35	P2	Ce5,2,3,4,6,7,8,10	1,5,9	2
31	314	33 516.43	34	P2	Ce3,2,3,4,6,8,10	1,5,7,9	2
32	296	31 275.81	31	P2	2,3,4,5,6,8,10	1,7,9	2
33	287	31 308.88	31	P2	1,2,3,4,10	5,6,7,8,9	2
34	274	30 292.60	30	P2	1,2,4,6,7,10	3,5,8,9	1
35	255	26 702.60	27	P1, P2	2,3,4,6s,7,8,10	1,5,9	2
36	250	26 532.38	27	P2	2,3,4,6,7,10	1,5,8,9	1
37	228	24 494.99	24	P2	2,4,5,6,8,10	1l,3,7,9	2
38	222	23 670.10	24	P2	2,4,6,7,8,10	1,3,5,9	2
39	195	20 767.84	21	P2	2,3,4,10	1,5,6,7,8,9	2
40	187	19 921.78	20	P1, P2	2,4,6s,7,8,10	1,3,5,9	2
41	173	18 263.93	18	P2	4,6,7,8,10	1,2,3,5,9	2

Protein length is represented by the number of amino acid residues (aa). The molecular mass of each isoform is indicated in Da and kDa. The *MBNL1* P1–P3 promoters from which the indicated isoforms originate, as well as the translation stop sites (Transl. Stop), are indicated (see also Fig. 1). The exonic composition, including coding exons and missing (Δ) MBNL1 exons 1–9, is specified for each isoform. Ce indicates cryptic exons. The short alternative variant of exon 6 is designated 6s. 1/1l indicates either the complete absence of exon 1 (1) or the absence of the full-length exon 1 (1l) concomitant with the presence of a short noncoding variant of exon 1. The main isoforms (40-43 kDa) are shown in bold, and the MBNL1 isoform 1 (Δ5,Δ9) was selected for sequence alignment in Fig. 2A. The table was prepared based on the RefSeq data available in the UCSC Genome Browser (GRCh38/hg38) and NCBI Gene.

**Table 2.** Human MBNL2 and MBNL3 protein isoforms

MBNL2							
Isoform	Length (aa)	Mol Mass (Da)	kDa	Promoters	Coding exons	ΔExons (1–9)	Transl. Stop
1	367	39 334.10	39	P1, P2	1,2,3,4,6,7,8,10	5,9	2
3	361	39 355.91	39	P1, P2	1,2,3,4,6,10	5,7,8,9	3
4	373	40 517.29	41	P1, P2	1,2,3,4,6,7,10	5,8,9	3
5	385	41 276.34	41	P1, P2	1,2,3,4,5,6,7,8,10	9	2
6	379	41 298.15	41	P1, P2	1,2,3,4,5,6,10	7,8,9	3
7	355	38 172.72	38	P1, P2	1,2,3,4,6,8,10	5,7,9	2
8	273	29 773.05	30	P1, P2	1,2,3,6,10	4,5,7,8,9	3
9	272	29 637.13	30	P2	1,2,3,4,10	5,6,7,8,9	2
10	391	42 459.53	42	P2	1,2,3,4,5,6,7,10	8,9	3
11	383	41 047.10	41	P2	1,2,3,4,5s,6,7,8,10	9	2
12	373	40 114.96	40	P2	1,2,3,4,5,6,8,10	7,9	2
13	353	38 591.53	39	P2	1,2,3,4,6,9,10	5,7,8	1
14	346	37 693.19	38	P2	1,2,3,4,6,8s,10	5,7,9	1
15	332	35 550.81	36	P2	1,2,3,4,6s,7,8,10	5,9	2
16	330	35 604.62	36	P2	Ce1,2,3,4,6,7,10	5,8,9	3
17	324	34 421.43	34	P2	Ce1,2,3,4,6,7,8,10	5,9	2
18	318	34 443.24	34	P2	Ce1,2,3,4,6,10	5,7,8,9	3
19	312	33 260.05	33	P2	Ce1,2,3,4,6,8,10	5,7,9	2
20	306	33 113.64	33	P2	1,3,4,6,10	5,7,8,9	3
21	303	32 876.67	33	P2	1,2,3,5,6,7,10	4,8,9	3
22	291	31 715.29	32	P2	1,2,3,5,6,10	4,7,8,9	3
23	266	28 586.63	29	P2	2,3,4,6,10	1,5,7,8,9	3
24	218	23 530.78	24	P2	1,3,6,10	2,4,5,7,8,9	3
25	272	28 564.81	29	P2	2,3,4,6,7,8,10	1,5,9	1
**MBNL3**							
**Isoform**	**Length (aa)**	**Mol Mass (Da)**	**kDa**	**Promoters**	**Coding exons**	**ΔExons (1-8)**	**Transl. Stop**
1	354	38 531.45	39	P1, P2	1,2,3,4,5,6,7,9	8	2
2	334	36 379.88	36	P3	1,2,3,4,5s,6,7,8	-	1
5	258	27 687.01	28	P1, P2, P4	2,3,4,5,6,7,9	1/1l,8	2
6	369	40 164.26	40	P1, P2	1,2,3,4,5,6,7,8	-	1
7	319	34 747.08	35	P1, P2	1,2,3,4,5s,6,7,9	8	2
8	273	29 319.82	29	P1, P2, P4	2,3,4,5,6,7,8	1	1
9	261	28 151.41	28	P1	2,3,4,5,7,8	1,6	1
10	246	26 518.60	27	P1, P2, P4	2,3,4,5,7,9	1,6,8	2
11	236	25 216.09	25	P1	3,4,5,6,7,9	1,2,8	2
12	223	23 902.64	24	P1	2,3,4,5s,6,7,9	1,8	2
13	167	17 567.55	18	P1	2,3,5,7,9	1,4,6,8	2

Protein length is represented by the number of amino acid residues (aa). The molecular mass of each isoform is indicated in Da and kDa. The *MBNL2* P1–P2 and *MBNL3* P1–P4 promoters from which the indicated isoforms originate, as well as the translation stop sites (Transl. Stop), are indicated (see Fig. 1). The exonic composition, including coding exons and missing (Δ) MBNL2 exons 1–9 and MBNL3 exons 1–8, is specified for each isoform. Ce indicates cryptic exons. The short alternative variants of exon 5 (MBNL2/3) and exons 6 and 8 (MBNL2) are designated 5s, 6s, and 8s, respectively. 1/1l indicates either the complete absence of exon 1 (1), or the absence of full-length exon 1 (1l) concomitant with the presence of a short noncoding variant of exon 1. The MBNL2 isoform 1 (Δ5,Δ9) and MBNL3 isoform 1 (Δ8) were selected for sequence alignment in Fig. 2A. The table was prepared based on the RefSeq data available in the UCSC Genome Browser (GRCh38/hg38) and NCBI Gene.

*MBNL* exons e1, e2, and e4 encode ZnF domains arranged in the tandem motifs ZnF1–2 and ZnF3–4 (Figs 1 and 2A). While ZnF1 and ZnF3–4 are encoded entirely by e1 and e4, respectively, the coding sequence of ZnF2 is split between e1 and e2, with the latter covering its major part [2] (Fig. 1). Both ZnF pairs are juxtaposed and joined via flexible and unstructured linker encoded by e3, which connects the ZnF2 and ZnF3 domains and is essential for splicing regulatory activity [34–36] (Figs 1 and 2A). However, the presence of other alternative exons may also influence the structure of MBNLs, the arrangement of ZnFs and hence the binding to target mRNAs (Tables 1 and 2, and Fig. 2B).

The sequences within e2, e3, and e6 of *MBNL1* contain proline-rich motifs that enable interactions with the Src-homology 3 (SH3) domains present in many signaling proteins, including Src family kinases [37]. Exon 5 and the highly conserved five amino acid KRAEK motif in e6 encode a bipartite nuclear localization signal (NLS) [35, 38] (Figs 1 and 2A). Although the KRAEK motif alone is sufficient for the nuclear redistribution of *Drosophila* Mbl [38], analyses of human MBNL1 truncation mutants demonstrated that in fact both e5 and e6 are required for exclusive nuclear retention [35]. Indeed, the lack of the full NLS in *Mbnl3* promoted its cytoplasmic localization, despite the presence of the KRAEK motif [25]. The C-terminus, particularly the sequences encoded by e7, determines homotypic MBNL–MBNL interactions and dimerization as well as improved binding of the protein to the CUG repeated region [35].

### Structural features of MBNL proteins essential for target recognition and splicing

MBNL ZnFs are composed of three cysteines followed by one histidine (CCCH) arranged in one (invertebrates) or two (vertebrates) similar compact pairs, i.e. tandem motifs (Fig. 2A and B). Notably, vertebrate MBNL proteins share 99% identity within the ZnF domains architecture and structure [39] (Fig. 2A). The ZnF1–2 tandem is positioned toward the N-terminal end of the protein, whereas ZnF3–4 is arranged within its middle part (Fig. 2B). Both pairs and each individual ZnF are separated from each other by amino acid linkers. While the linker connecting the two individual ZnFs within each tandem orients their RNA-binding surfaces in a back-to-back position, enforcing an antiparallel alignment of bound RNA strands ([39] and reviewed in [2]), the unstructured linker separating the two tandems supports protein flexibility and facilitates a wide array of interactions with differentially structured RNAs [34, 35, 39, 40]. Consistently, its loss dramatically decreases the affinity of MBNL for target sequences [35, 36].

Mutagenic studies of ZnFs revealed that while disruption of either individual or tandem ZnF only marginally affected MBNL1 splicing activity, it almost completely abrogated it when combined and arranged pairwise to affect both ZnF pairs [41]. Although this finding supports a model in which either ZnF tandem is sufficient for splicing regulation, mutations ablating specific RNA interactions via individual ZnFs revealed that the splicing activities of ZnF1–2 and ZnF3–4 were not equivalent for all classes of splicing events. While their redundancy was observed for some pre-mRNA targets, a subset of MBNL1-regulated events required functional ZnF1–2 for effective splicing regulation [41].

Consistently, studies with chimeric MBNL proteins carrying duplicate ZnF1–2 or ZnF3–4 demonstrated that duplication of the former tandem strikingly increased splicing activity compared with the canonical arrangement, whereas duplication of the latter markedly decreased it because of the partial loss of the ability to recognize canonical target motifs [42]. A model emerging from these studies proposed that both domains act as independent units: while ZnF1–2 confers specific sequence recognition, ZnF3–4 acts as a more general RNA-binding domain [42]. In agreement with these findings, targeted point mutations interfering with the RNA binding of MBNL1 ZnF1–2 were more detrimental for splicing activation, repression and RNA binding than those in ZnF3–4 [40].

### MBNL consensus motif

MBNLs recognize a consensus GC motif embedded in pyrimidines (YGCY motif, where Y is a pyrimidine C or U) with a strong preference for the UGCU motif [9, 18, 43–45]. In particular, CUG and CCUG repeated sequences expanded in mutated transcripts found in DM types 1 (DM1) and 2 (DM2), respectively, constitute high-affinity binding sites for MBNLs [2, 46]. Target recognition mechanisms via ZnFs are highly conserved; *Drosophila* Mbl ZnFs can bind human MBNL1 targets [47] and even regulate the AS of murine reporter minigenes [48].

Although all mammalian MBNL paralogs recognize and bind the same RNA motifs and often regulate the splicing of the same exons, they do so with different affinities and different strengths, with MBNL1 possessing the strongest and MBNL3 the weakest splicing activity [49]. Because the ZnF-encoding amino acid sequence is largely conserved among MBNL paralogs (Fig. 2A), these differences likely result from alternative exon usage (Tables 1 and 2), variations in nucleoplasmic and cytoplasmic distribution, and differences in the structural context of target RNA sequences and motif distribution, including higher-order motif clustering of YGCY elements [35, 49–51].

### Multiple layers of RNA processing by MBNL proteins

Among the multifaceted functions of MBNLs in RNA processing, AS constitutes one of their most prominent roles. While the main type of AS regulation involves the promotion of the skipping or inclusion of alternative cassette exons, these RBPs also control the selection of mutually exclusive exons, shift exon boundaries by choosing alternative 3′ and 5′ splice sites (3′ss and 5′ss, respectively), and suppress or promote intron retention [44, 52]. Beyond this, MBNLs may select alternative last exons and alternative polyadenylation sites, influencing the length of the 3′UTR, transcript stability and localization. Other roles of MBNLs in RNA processing include miRNA biogenesis and circRNA formation ([53] and recently reviewed in [54]). Collectively, these activities enable MBNL proteins to shape the composition and function of the cellular transcriptome.

### Positional model of MBNL splicing activity

**Figure 3.**
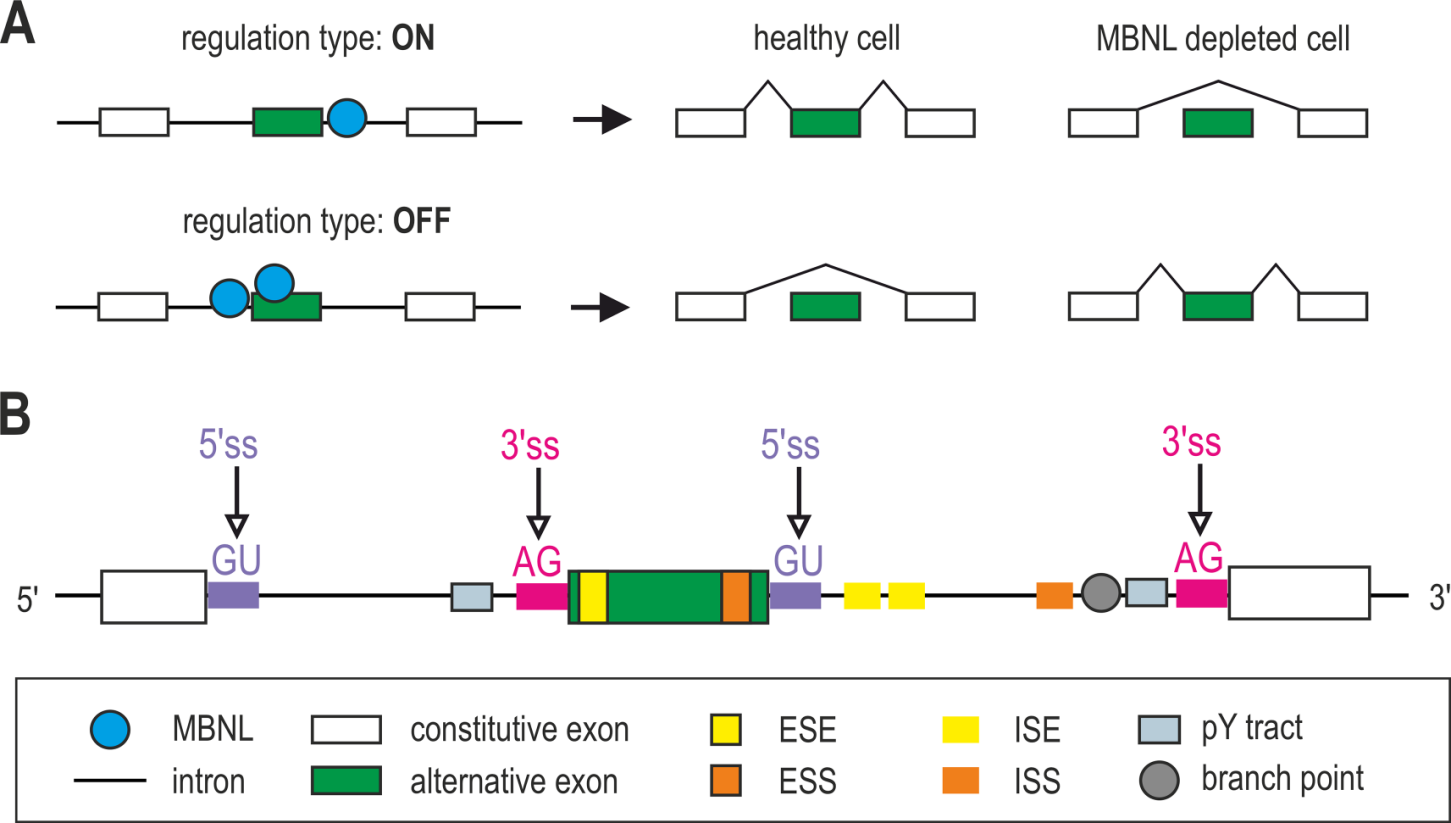
Positional model of MBNL-dependent AS regulation. (**A**) Sequence- and position-dependent binding of MBNL proteins to their cognate motifs controls exon inclusion (“ON” regulation) or exclusion (“OFF” regulation). The pattern present in healthy cells is reversed in cells with MBNL deficiency, such as DM cells. (**B**) Major molecular mechanisms involved in MBNL-mediated exon inclusion or repression. Further details are provided in the main text.

MBNLs modulate AS events by promoting or suppressing exon inclusion in mature transcripts via sequence- and position-dependent binding to cognate motifs within target pre-mRNAs, relative to the regulated exon [6, 43, 44, 45, 55]. As a general rule, MBNLs enhance exon inclusion when bound near the downstream exon/intron junction (defining the 5′ss - splice donor at the beginning of an intron) and suppress exon inclusion by binding near the upstream intron/exon boundary of the regulated exon (defining the 3′ss - splice acceptor at the end of an intron) or within the regulated exon ([43–45] and reviewed in [2, 55]) (Fig. 3A). This positional mode of action resembles splicing regulation by other splicing factors including CELF, FOX, NOVA, PTB or SR, and hnRNPL (reviewed in [56]).

Mechanistically, the promotion of exon inclusion may follow MBNL-dependent activation of intronic splicing enhancers (ISEs) or blocking of intronic or exonic splicing silencers (ISS and ESS, respectively), the latter usually located within the proximity of the 3′ end of an exon. Furthermore, enhanced recognition of 5′ss by spliceosome, or MBNL competition with trans-acting splicing silencers may also enhance MBNL-dependent exon inclusion. Conversely, MBNL-mediated splicing inhibition may result from the blocking of *cis-*acting sequence motifs, including 3′ss, polypyrimidine tracts (pY tracts) or intronic branch site elements as well as exonic splicing enhancers (ESEs) located within the 5′ end of an exon. Alternative exon repression may also result from direct competition with essential splicing factors [57, 58] (Fig. 3B).

### Alternative splicing of *MBNL* exons

*MBNL* pre-mRNAs undergo multiple AS events that shape their expression levels, isoform diversity, subcellular localization, and tissue-specific functions. More importantly, many exons, including e1, e3, e5, e7, and e8, are controlled by MBNL-dependent negative feedback loops, adding an additional regulatory layer of isoform composition adjustment through the AS of own pre-mRNAs ([24, 59] and reviewed in [23]).

For example, autoregulated skipping of the first coding exon (e1) of *MBNL1*, triggered by all MBNL paralogs through binding to cognate motifs in the 5′UTR constituting part of e1, critically diminishes the stability and splicing activity of the resulting protein. This occurs because skipping of e1 shifts translation initiation to e2, thereby bypassing and eliminating the ZnF1–2 domain [23, 24]. Consequently, repression of e1 is released, enabling e1-containing RNAs to resume translation of the full-length, functional MBNL1 isoforms.

The e1-loop buffers the cellular pool of MBNL1 and is active in DM1, providing partial protection against MBNL sequestration by toxic expanded RNAs, although this buffering capacity is progressively exhausted as repeat length increases and disease severity advances [24]. In addition, this regulatory circuit relies strongly on promoter-specific transcription, as predominantly P2-derived *MBNL1* pre-mRNAs exhibit MBNL-dependent AS of e1 [23, 24].

While short isoforms devoid of e1 have been described for human *MBNL1* and murine *Mbnl1* as well as for *Mbnl2*, they are poorly translated and give rise to highly unstable proteins [6, 19, 23, 24], possibly due to inefficient loading of mRNAs lacking e1 on polysomes [24]. Conversely, the production of a stable isoform lacking e1 and devoid of ZnF1–2 may be unique to eutherian-specific MBNL3 paralog [7, 25, 60].

A further example is the repression of e5, which adjusts the distribution of MBNL1 and MBNL2 protein isoforms between the nucleus (+e5) and the cytoplasm (-e5), influencing their activity and target selectivity [38, 59]. Likewise, data from worm as well as mammalian models show that the negative feedback loop through e7 splicing may alter the structural flexibility of the C-terminus, affecting MBNL protein dimerization and multimerization, binding properties and subcellular distribution to some extent [61, 62]. These selected examples underscore how feedback-based splicing circuits shape MBNL protein isoform balance and cellular behavior, which is critical in response to constantly changing environmental conditions.

### Interplay of MBNLs with other RBPs

MBNLs function within a broad network of cooperating and competing RBPs, which collectively shape RNA processing outcomes [56]. Hence, the amount of MBNLs relative to other splicing factors affects both the direction and the strength of the splicing response. These dynamic changes are critical throughout development, when many RBPs opposing MBNLs are highly expressed in fetal tissues and decline postnatally while MBNLs rise, allowing for developmental transition of the AS program.

One example involves functional antagonism between MBNLs and CELF1/2, which may bind overlapping or adjacent motifs on many shared targets [63]. This is also evident in DM1, where an insufficiency of MBNLs and an increase in the steady-state levels of CELF1 due to its hyperphosphorylation via excessively active protein kinase C (PKC) synergistically shift the AS toward fetal-like pattern [64].

Depending on the context, MBNLs may cooperate or compete with core spliceosomal components and auxiliary RBPs including RBFOX [65], NOVA [44], PTBP1/2 [66, 67], hnRNPH [68], and SR proteins (e.g. SRSF1 [69]), contributing to combined splicing outcomes. While in some cases such an interplay may collectively promote similar isoforms and stabilize each other’s binding at weak sites, in others, it leads to competition at overlapping regulatory regions of pre-mRNAs.

### MBNL dosage effect

The overall amount of available MBNL protein pool largely determines the strength of exon exclusion versus inclusion. Studies in human myoblasts with siRNA-mediated incremental depletion of the MBNL pool demonstrated a dose-dependent effect on the number and severity of the AS defects [70]. Furthermore, precise tuning of MBNL1 in a cellular model revealed that splicing events exhibit different responses depending on the amount of protein available, and indicated the critical role of the number, location and affinity of the MBNL1-binding sites [71]. In addition, engineered, inducible expression of exogenous MBNL1 revealed that MBNL1-regulated exclusion events require higher concentrations of the protein to properly regulate AS outcomes than inclusion events do [65]. Considering that MBNL dose variations have an important effect on DM variability, these dose-dependent MBNL-regulated AS events are excellent biomarkers of the disease severity and response to therapeutics [71, 72].

## Animal models of MBNL deficiency

Genetically modified animal models have been invaluable for deciphering the roles of individual MBNL proteins in physiological and molecular processes. We summarize key lessons from these models focusing primarily on murine *Mbnl* loss-of-function (LOF) mutants (Table 3) and non-mammalian vertebrate model zebrafish *zmbl* mutants. We also highlight selected studies in invertebrates including nematodes and fruit flies. Owing to discrepancies in *Mbnl/MBNL* exon nomenclature across the literature, we use the first coding exon (e1), shown in Fig. 1, as a reference when describing mouse mutants.

**Table 3.** Selected examples of murine *Mbnl* LOF mutants

*Mbnl* gene mutant	Mouse model	RNA and protein expression	Key phenotype features and findings	Ref.
*Mbnl1* KO	*Mbnl1* ^ΔE3/ΔE3^(deletion of the first coding exon by homologous recombination)	- Partial decrease of *Mbnl1* mRNA (some isoforms present); - No expression of MBNL1 protein; - Compensatory increase in MBNL2 protein	- Myotonia, myopathy, cataracts;- Aberrant AS; - No muscle weakness; - No AS dysregulation in the brain; - Postnatal thymic hyperplasia with RNA misprocessing [127]	[19]
*Mbnl1* KO	*Mbnl1* ^ΔE2/ΔE2^(germline deletion of loxP flanked exon harboring translation start, via Prm-Cre)	- Absence of mRNA and protein; - A compensatory (2.5-fold) increase in the steady-state levels of MBNL2 protein	- Short lifespan; sudden death between 4.8–6.5 months of age; - Cardiac pathology (elevated heart rates, heart hypertrophy, fibrosis, and calcification); - Persistence of embryonic splice isoforms in mutant hearts	[75]
*Mbnl2* GT	*Mbnl2* ^GT4/GT4^(GT insertion in intron 4)	- 90% reduction of *Mbnl2* mRNA; - Mutant encodes a truncated protein with two ZnFs	- No DM phenotype; - No apparent contribution of MBNL2 loss to DM muscle pathology; - No AS alterations	[5]
*Mbnl2* GT	*Mbnl2* ^GT2/GT2^(GT insertion in intron 2)	- *Mbnl2* RNA significantly decreased; - Truncated MBNL2 protein with only one complete ZnF detected (a fusion molecule encoded by the first coding exon fused in-frame to a neo-reistance/βgeo-reporter)	- Independent role of MBNL2 in mature skeletal muscle; - Skeletal muscle pathology (decreased myofiber size, central nuclei, and fibrosis); - Myopathy; - AS defect of calcium chloride channel but no alterations in splicing of other genes; - Late onset myotonia	[76]
*Mbnl2* KO	*Mbnl2* ^ΔE2/ΔE2^(removal of the first coding exon by homologous recombination)	- Ablation of full-length *Mbnl2* mRNA and absence of protein; true functional null mutants; - Upregulation of MBNL1 protein in *Mbnl2* KO brain	- No overt skeletal muscle pathology or myotonia; - Essential role of MBNL2 in brain development and hippocampal synaptic plasticity; - CNS pathologies with widespread AS defects in the KO brain; - REM sleep propensity, seizures and spatial memory deficits	[6]
*Mbnl2* Cond. KO	*In utero* electroporation of Cre recombinase into the developing cortex of *Mbnl2*^f/f^ mice	- Ablation of *Mbnl2* expression in cortical neurons	- Decreased dendritic spine density and dynamics in adolescent mice; - *Add1* isoform switch from adult to fetal produced truncated ADD1 which failed to interact with SPTAN1, a critical protein for spinogenesis	[116]
*Mbnl3* KO	*Mbnl3* ^ΔE2/ΔE2^(removal of the first coding exon by homologous recombination)	- Absence of the full-length 38-kDa MBNL3 protein; - Upregulation of *Mbnl3* RNA and 27-kDa protein encoding short MBNL3 isoform with ZnF3-4 only, predominantly cytoplasmic	- No DM manifestation or overt muscle/CNS defects; minimal AS alterations [7]; - Progressive impairment of injury-induced skeletal muscle regeneration [7]; - Glucose intolerance, elevated insulin levels, cardiac dysfunction, and cataracts, largely w/o DM1-like AS defects [60]; - Accelerated onset of age-related pathologies [60]	[7, 60]
*Mbnl3* Cond. KO	*Mbnl3* ^condWL^ (Cond. null allele of *Mbnl3* with loxP sites flanking e2 and e7c); CMV-Cre	- Complete absence of *Mbnl3* RNA and protein (both long and short isoforms)	- Defective myoblast differentiation in culture and precocious expression of muscle-specific transcripts; - AS dysregulation in cultured myoblast; prevalent inclusion of adult pattern exons; - MBNL3 restricts placental growth: larger placentas in the absence of *Mbnl3* due to increased cell proliferation and upregulation of Myc and its associated regulatory network [25]	[52]
*Mbnl1/2* Combined DKO	*Mbnl1* ^ΔE3/ΔE3^ *Mbnl2* ^+/ΔE2^	- Homozygous DKO (*Mbnl1*^ΔE3/ΔE3^; *Mbnl2^ΔE2^*^/ΔE2^) are lethal; - DKO heterozygous for *Mbnl2* (*Mbnl1*^ΔE3/ΔE3^; *Mbnl2*^+/ΔE2^) show partial decrease of *Mbnl1* mRNA; - No expression of MBNL1 protein and compensatory increase of *Mbnl2* (RNA, protein) in the absence of *Mbnl1*	- Dual depletion worsens splicing defects and aggravates DM phenotype; - Severely reduced lifespan; - Muscle weakness/wasting, cardiac arrhythmia, and severe AS defects	[73]
*Mbnl1/2* Combined DKO (Cond.)	*Mbnl1* ^ΔE3/ΔE3^ (KO) / *Mbnl2*^c/c^; Hb9-Cre-MN (Cond. KO of *Mbnl2*)	- Full KO of *Mbnl1*; no expression of 41-kDa and 42-kDa proteins; - Neuromuscular junction-specific ablation of *Mbnl2*	- Gait coordination deficits associated with structural and ultrastructural defects in the neuromuscular junction; - AS aberrations in genes associated with synaptic transmission and neuromuscular junction homeostasis	[122]
*Mbnl1/2* CombinedDKO (Cond.)	*Mbnl1* ^ΔE3/ΔE3^ (KO) / *Mbnl2*^c/c^; Myo-Cre (Cond. KO of *Mbnl2*)	- Full KO of *Mbnl1;* no expression of 41-kDa and 42-kDa proteins; - Muscle-specific ablation of *Mbnl2*	- Profound deficiency of adult skeletal muscle; - Severe muscle pathology; - Aggravation of splicing defects	[73]
*Mbnl1/2* CombinedDKO (Cond.)	*Mbnl1* ^ΔE3/ΔE3^ (KO) / *Mbnl2*^c/c^; Myh6-Cre (Cond. KO of *Mbnl2*)	- Full KO of *Mbnl1*; no expression of 41-kDa and 42-kDa protein; - Cardiomyocyte specific ablation of *Mbnl2*	- Reduced lifespan; - Sudden death due to lethal cardiac rhythms; - Dilated fibrotic hearts; - Gene expression and AS changes recapitulating DM heart spliceopathy	[78]
*Mbnl2/3* Combined DKO	*Mbnl2* ^ΔE2/ΔE2^ (KO) / Mbnl3^condWL^; CMV-Cre (Cond. KO of *Mbnl3*)	- Absence of full-length *Mbnl2* mRNA and absence of protein; - Absence of *Mbnl3* RNA and protein (both long and short isoforms)	- Severe intrauterine growth restriction of DKO embryos; - Maturation defect in DKO placentas; - Large splicing changes in DKO placentas – *Mbnl2/3* coregulate placental AS and polyadenylation	[25]
*Mbnl1,2,3* Combined TKO (Cond.)	*Mbnl1* ^ΔE3/ΔE3^ (KO) /*Mbnl2*^c/c^; *Mbnl3*^condWL^; Myog-Cre (Cond. KO of *Mbnl2,3*)	- No expression of MBNL1 41-kDa and 42-kDa proteins; - Muscle-specific ablation of *Mbnl2/3*	- TKO exacerbates DM-like defects; - Severe myopathy and presence of congenital DM features	[52]

Abbreviations: KO, knockout; GT, gene trap; DKO, double knockout; TKO, triple knockout; Cond., conditional; c/c or f/f, conditional allele; WL, whole locus; Cond. KO, conditional KO; AS, alternative splicing; CNS, central nervous system; Ref., Reference publication. Owing to discrepancies in *Mbnl*/*MBNL* exon nomenclature across the literature, we use the first coding exon (corresponding to e1 shown in Fig. 1 for each *MBNL* paralog) as a point of reference when describing mouse mutants in the main text.

### *Mbnl1* loss-of-function mutant mice

*Mbnl1* isoform knockout (KO) mice with homologous recombination-mediated genetic ablation of the first coding exon harboring translation start (*Mbnl1^ΔE3/ΔE3^*; where E3 corresponds to e1 in human *MBNL1*, as shown in Fig. 1) recapitulated the DM phenotype including myotonia, myopathy, cataracts, and aberrant AS [19], but failed to develop cardiac pathology and muscle weakness [6, 19, 45, 73] or AS dysregulation in the brain [74] (Table 3).

Interestingly, germline-specific deletion of conditional alleles of *Mbnl1* (*Mbnl1^loxE2lox^*; where E2 corresponds to E3 in [19], and to e1 in human *MBNL1*, as shown in Fig. 1) significantly shortened the lifespan of mutant mice, leading to spontaneous death at approximately 6 months of age [75]. Mutant *Mbnl1^ΔE2/ΔE2^* animals presented overall cardiac pathology, including heart hypertrophy, calcification, and fibrosis. Furthermore, *Mbnl1* depletion caused embryonic splice isoforms to persist in a network of cardiac RNAs, including those implicated in DM [75].

### *Mbnl2* loss-of-function mutant mice

Gene trap (GT) studies have provided contradictory data on the effects of *Mbnl2* deficiency (Table 3). The first described *Mbnl2* mutant mice were generated by β-geo GT insertion into intron 4 of *Mbnl2* (*Mbnl2*^GT4/GT4^ mice; where intron 4 corresponds to the intron separating e3 and e4 in human *MBNL2*, as shown in Fig. 1) [5]. These mutants displayed no apparent changes in muscle structure or function and no AS alterations, in contrast to the *HSA*^LR^ (human skeletal actin long repeats) mouse model of DM, carrying ∼250 CTG repeats, or *Mbnl1* KO mice. Consistently, no effects on the development of DM features including myotonia were observed. However, the same GT inserted in intron 2 of *Mbnl2* (*Mbnl2*^GT2/GT2^ mice; intron 2 corresponds to the intron separating e1 and e2 in human *MBNL2* shown in Fig. 1) led to myotonia, skeletal myopathy and altered chloride channel expression characteristic of DM [76] (Table 3). These striking differences likely stem from GT insertion sites; while the former mutant contains additional coding regions yielding a truncated protein containing two ZnFs, the latter leads to the production of a molecule encoded by the first coding exon of *Mbnl2* fused in-frame to a β-geo reporter, yielding a truncated protein with only one complete ZnF. Moreover, the late onset myotonia phenotype in *Mbnl2*^GT2/GT2^ mice [76] is consistent with the adult-onset phenotype often observed in DM patients, indicating that MBNL2 may play an independent role in mature or remodeling skeletal muscle that is not completely compensated for by the presence of MBNL1 [76].

To address these inconsistencies, LOF *Mbnl2* mutant mice, lacking the first coding exon harboring the initiation codon for the full-length MBNL2 protein (*Mbnl2*^ΔE2/ΔE2^; where E2 corresponds to e1 in human *MBNL2*, as shown in Fig. 1), were generated via homologous recombination [6]. Removal of the first coding exon led to ablation of the full-length mRNA and a complete absence of the MBNL2 protein, indicating that these mutants were true functional nulls (Table 3). Strikingly, *Mbnl2*^ΔE2/ΔE2^ mice did not develop any apparent skeletal muscle pathology or motor deficits but instead presented phenotypes consistent with pathologies observed in the CNS of DM patients, including widespread splicing abnormalities in the brain, increased REM sleep propensity and seizure susceptibility, as well as deficits in spatial memory [6]. MBNL2 expression is prominent in the hippocampus, and *Mbnl2*^ΔE2/ΔE2^ KO animals presented impaired hippocampal synaptic plasticity associated with a decrease in *N*-methyl-d-aspartate receptor (NMDAR) synaptic transmission. Taken together, these data suggest that while MBNL2 alone does not significantly contribute to DM skeletal muscle pathology, it plays a major role during postnatal brain development [6], in accordance with its predominant expression in this tissue [2].

### *Mbnl3* loss-of-function mutant mice

Spatial and temporal analyses of the *Mbnl3* expression pattern during murine development have indicated that it peaks early in embryonic life and is largely absent or low in adult tissues [7, 77]. In contrast to the other paralogs, *Mbnl3* mutants generated by homologous recombination of the first coding exon (*Mbnl3^ΔE2/ΔE2^* mice; E2 corresponds to e1 in human *MBNL3*, as shown in Fig. 1), which eliminates the full-length MBNL3 protein but retains the shorter isoform with ZnF3–4, fail to develop overt muscle- or CNS-related phenotypes during postnatal development [7, 60] (Table 3). Instead, a progressive decline in skeletal muscle regenerative capacity, manifested as an age-dependent delay in myotoxin-induced muscle injury/regeneration, has been demonstrated in these mice [7].

In addition to impairing muscle regeneration, MBNL3 deficiency accelerated the onset of a subset of age-associated DM-like pathologies including glucose intolerance and elevated insulin levels, cardiac dysfunctions, and cataracts [60]. Intriguingly, these changes were accompanied by minimal AS alterations, suggesting that mechanisms distinct from embryonic splice isoform retention may contribute to the onset of age-associated phenotypes in DM [7, 60].

To ablate both long and short *Mbnl3* isoforms, conditional mouse mutants were generated by removing loxP-flanked exons 2 (the first coding exon) and 7c [52]. Multiple transcriptome abnormalities accompany MBNL3 deficiency, including early expression of muscle-specific transcripts in knockout myoblasts as well as preferential inclusion of adult pattern exons in many misregulated AS events [52]. In another study, these mutants presented significantly increased placental weight throughout embryonic development, suggesting that MBNL3 plays a role in restricting placental growth [25].

### Compound loss of mammalian *Mbnl* genes

Combined *Mbnl* KO mutant mice display more severe phenotypes than single KO, implying functional compensation of MBNL proteins (Table 3). While *Mbnl1* and *Mbnl2* double KO (DKO) homozygous mutant mice (*Mbnl1^ΔE3/ΔE3^; Mbnl2^ΔE2/ΔE2^*) are embryonic lethal, inactivation of both alleles of *Mbnl1* and only one copy of *Mbnl2* (heterozygous *Mbnl1^ΔE3/ΔE3^; Mbnl2^+/ΔE2^* mutant mice) is compatible with survival but dramatically reduces lifespan and body weight, with severe progressive muscle weakness and wasting, cardiac arrhythmia, and DM-like splicing defects [73]. The dual depletion of *Mbnl1* and *Mbnl2* also exacerbated the splicing alterations observed in *Mbnl1* KO animals [73].

Similarly, conditional DKO mice with *Myo-Cre*-mediated muscle-specific elimination of *Mbnl2* in an *Mbnl1* KO background presented dramatic neonatal lethality and body weight reduction at birth, along with a profound deficiency of adult skeletal muscle and severe muscle pathology. These conditional DKOs presented more pronounced AS defects than *Mbnl1* KO alone did [73].

Most recently, *Myh6-Cre*-mediated conditional deletion of *Mbnl2* in cardiomyocytes in an *Mbnl1* KO background resulted in sudden death due to spontaneous lethal cardiac rhythms [78]. Studies with muscle-specific double (*Mbnl1* and *2*) and triple (*Mbnl1, 2*, and *3*) conditional KO mouse models demonstrated that compound deficiency of MBNLs aggravated these defects and recapitulated many of the phenotypes associated with congenital forms of DM, including myopathy and associated widespread mis-splicing events [52].

### Compensatory roles of MBNL paralogs revealed by loss-of-function studies

Functional compensation between MBNL paralogs allows the loss of one paralog to be counterbalanced by increased expression of the remaining paralog, helping to maintain normal RNA processing and AS patterns. Compensation is context- and tissue-dependent and is based on the shared RNA-binding specificity as well as the subcellular localization of the paralogs.

**Figure 4.**
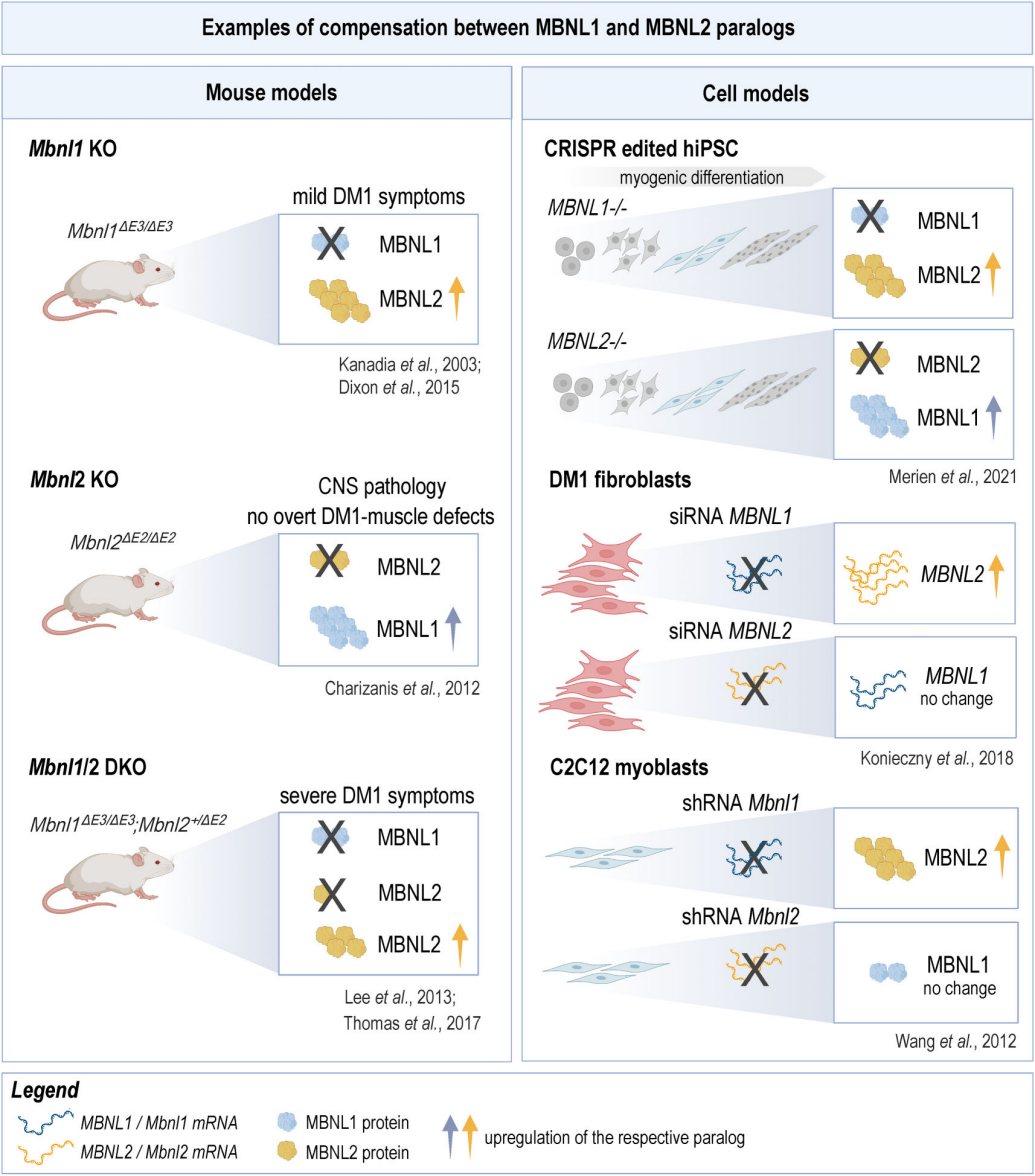
Examples of compensation between the MBNL1 and MBNL2 paralogs. Left panel: Compensatory upregulation of the MBNL2 or MBNL1 protein in the absence of the other paralog, respectively, was reported in *Mbnl1* KO [19, 75], *Mbnl2* KO [6] and *Mbnl1/2* DKO [52, 73] mouse models. Right panel: Similar compensation, either at the RNA or protein level, was observed in distinct human and murine cell models. These include CRISPR-edited hiPSC without *MBNL1* or *MBNL2* and undergoing myogenic differentiation [80], DM1 fibroblasts [23] and C2C12 myoblasts with *MBNL1* knockdown [44]. Relative upregulation levels are represented by the number of protein and RNA molecules; wild-type levels are represented by two molecules. The legend is shown. More details are provided in the main text. Figure created in BioRender [Stepniak-Konieczna, E. (2026), https://BioRender.com/ej75chg] and modified in CorelDraw.

An increase in the MBNL2 protein in the absence of MBNL1 is one of the best studied examples of such compensation, as documented in distinct mouse and cell models (Fig. 4). In *Mbnl1* KO mice, consistent upregulation of the MBNL2 protein partially maintains the splicing of shared target exons, although many MBNL1-specific targets remain misspliced [19, 75]. Similarly, the protein levels of MBNL1 were elevated in *Mbnl2* KO mice but only in the brain [6]. Interestingly, *Mbnl1* KO mice deficient in one allele of *Mbnl2* displayed much more severe DM1 symptoms than did mice lacking MBNL1 alone, suggesting that MBNL2 may be involved in modifying DM1 severity [52, 73]. These mice also presented elevated MBNL2 protein levels compared with WT mice [73]. Recent work has shed more light on the mechanism of compensatory upregulation of MBNL2 and demonstrated that it is based on the autoregulatory splicing of *Mbnl2* exons by MBNL1 [79]. Specifically, the loss of MBNL1 promoted the inclusion of e6 and e9 in *Mbnl2* mRNA (corresponding to e5 and e8, respectively, in *MBNL2*, as shown in Fig. 1). While e6 inclusion increased MBNL2 translocation to the nucleus, e9 inclusion shifted the reading frame to an alternative C-terminus lacking the PEST domain required for proteasomal degradation, hence positively affecting the protein stability [79]. Interestingly, according to UCSC Genome Browser and NCBI RefSeq data, the presence of either e8 or e9 in human *MBNL2* is concomitant with the inclusion of a shorter variant of e10 lacking the PEST domain (see also Fig. 1), suggesting that similar mechanisms may operate in humans.

Compensatory mechanisms have also been documented in cell models, including CRISPR-edited human-induced pluripotent stem cells (hiPSCs) differentiated into skeletal muscle [80]. Here, the MBNL1 protein level was elevated in *MBNL2-*deficient cells; conversely, the MBNL2 level increased in *MBNL1-*deficient cells, whereas the MBNL3 level remained unaffected by the loss of either paralog [80]. Enhanced *MBNL2* mRNA levels were reported in DM1 fibroblasts with siRNA-mediated knockdown of *MBNL1*, but not vice versa [23]. Similarly, MBNL2 protein levels were upregulated following shRNA-mediated knockdown of *Mbnl1* in C2C12 myoblasts; however, the reciprocal effect was not observed [44].

What is the biological significance of MBNL1 and MBNL2 compensation? Available data indicate that it stabilizes AS by buffering fluctuations in protein levels and preventing widespread mis-splicing. This is supported by dramatically worsened splicing defects in *Mbnl1/2* DKO compared with *Mbnl1* KO mice [73] and is clearly disease relevant, as the compensatory mechanism between the paralogs could be leveraged for therapeutic purposes.

### Zebrafish *muscleblind* mutants

Three *muscleblind-like* genes (*zmbnl1, zmbnl2*, and *zmbnl3*) have been identified in the zebrafish genome [12]. Although all of these genes share similar genomic organization and give rise to primary transcripts undergoing extensive AS, comparable to human *MBNL* genes, several key differences exist between zebrafish and mammalian *muscleblind* orthologs.

First, AS of the first three exons in *zmbnl2*, but not *zmbnl1* or *zmbnl3*, generates mRNAs translated into a protein lacking the first ZnF pair [12]. Notably, alternative transcript isoforms with the potential to encode a short protein devoid of ZnF1–2 have been described for *MBNL1* as well as *Mbnl1* and *Mbnl2* but appear to produce unstable proteins [6, 19, 24] unless stabilized by GST or GFP tags [24, 50, 81]. Conversely, a stable isoform devoid of the first ZnF pair was detected for MBNL3 [7] and may be unique to placental mammals [25]. Second, all the *zmbnl* genes, including *zmbnl3*, are expressed in adult zebrafish tissues, which is in stark contrast to orthologs from higher vertebrates such as mice and humans, in which *MBNL3* is expressed at a very low level in most adult tissues [12]. Third, unlike in mammals, zebrafish *zmbnl1* is not expressed predominantly in the skeletal muscle or heart. While *zmbnl1* is expressed in most adult tissues, *zmbnl2* is found mainly in the heart and muscle, whereas *zmbnl3* is expressed in the eye, muscle, heart and testis [12].

The first comprehensive zebrafish model of DM-associated MBNL depletion was generated through LOF mutation of the three zebrafish z*mbnl* genes [13]. Homozygous zebrafish single, double, and triple z*mbnl* LOF mutants are viable to adulthood, which is clearly different from the phenotype observed in mouse mutants, in which combined loss of *Mbnls* in a homozygous state is incompatible with survival [52, 73]. Triple *zmbnl* zebrafish mutants present phenotypes that are consistent with those observed in DM patients, e.g. decreased body size, altered motor function and widespread changes in AS events [13].

### Nematode models of *muscleblind* deficiency

The nematode *Caenorhabditis elegans* contains a single *muscleblind* (*mbl-1*) gene orthologous to the mammalian *MBNL* [82]. The *Mbl-1* gene was initially shown to generate two transcript isoforms, *CeMbl-a* and *CeMbl-b* [11, 21]. In contrast to mammalian MBNLs, CeMBL-A and CeMBL-B proteins contain only two ZnFs with a distinguishable CCCH domain [11, 82], highly homologous to the CCCH ZnF found in human MBNL1 [82]. Although both *mbl-1* transcripts are detectable in the larval and adult stages, they seem to play major roles in an adult organism [11].

In early studies, full-genome RNA interference (RNAi) knockdown of the predicted *C. elegans mbl-1* gene did not affect embryogenesis at all, suggesting MBL-1 redundancy in early embryo and larval development [83]. Similarly, *mbl-1* RNAi affects only muscle structure and function in adult worms [11]. MBL-1 has also been demonstrated to play essential roles in neuromuscular junction synapse formation in neurons, dendrite morphogenesis [84, 85], and the regulation of nematode lifespan [86, 87].

The *mbl-1* mutant with deletion of e3 and flanking introns, resulting in a protein that lacks the ability to bind RNA, presented a reduced lifespan similar to that of the *exc-7;mbl-1* double mutant [82]. More recent analyses of the reduced lifespan phenotype revealed that *mbl-1* deficiency in mutant worms reduced the activity of p38 mitogen-activated protein kinase (MAPK; PMK-1 in *C. elegans*) and its downstream target genes *atf-7* and *skn-1* [88].

### *Drosophila muscleblind* mutants

In the fruit fly *Drosophila melanogaster*, a single *muscleblind* gene (*mbl*) gives rise to a primary transcript that undergoes complex AS to generate seven mature canonical transcripts encoding the protein isoforms MblA-G [55, 89]. Isoforms *mblA-D* have been most extensively studied. Whereas *mblA-C* are translated into proteins with two complete CCCH-type ZnFs, *mblD* generates a smaller protein with only one complete ZnF [9, 21]. Profiling of *mbl* isoforms during fruit fly development revealed that they are functionally distinct, with *mblC* being the most widely expressed as well as the most ancient isoform [48, 90].

The first described *Drosophila mbl* LOF lethal mutants lacked normally differentiated photoreceptors [9]. Further work revealed that the lethal *mbl* LOF mutants die as late embryos with a clear musculature phenotype involving disruption of sarcomeric Z bands and a lack of extracellular matrix at indirect muscle attachment sites [10]. Muscle defects in *mbl* mutants are apparently caused by AS alterations in multiple muscle-specific transcripts, including those encoding α-actinin [90, 91], troponin T [48] and calcium pump dSERCA [92].

Notably, among all the isoforms, only MblC overexpression (OE) was able to rescue most of the *mbl* LOF lethal mutant embryos [90]. Remarkably, human *MBNL1* expressed in *Drosophila* Mbl-deficient embryos also rescued embryonic lethality [93] and suppressed both the somatic muscle and rough eye phenotypes in the *Drosophila* DM1 model i(CTG)480 (producing a noncoding mRNA containing 480 interrupted CUG repeats) [94, 95]. These results indicate functional conservation between human and fly proteins. Conversely, a reduction in muscleblind levels caused by a heterozygous LOF mutation in *mbl* aggravated the i(CTG)480 eye phenotype [94]. While the degree of i(CTG)480 phenotype suppression depended on MBNL1 OE levels, transgenic lines expressing high levels of MBNL1 exhibited a muscle phenotype on their own, even in the absence of i(CTG)480 [94], highlighting the importance of maintaining proper steady-state protein levels.

## MBNL proteins in physiological processes

**Figure 5.**
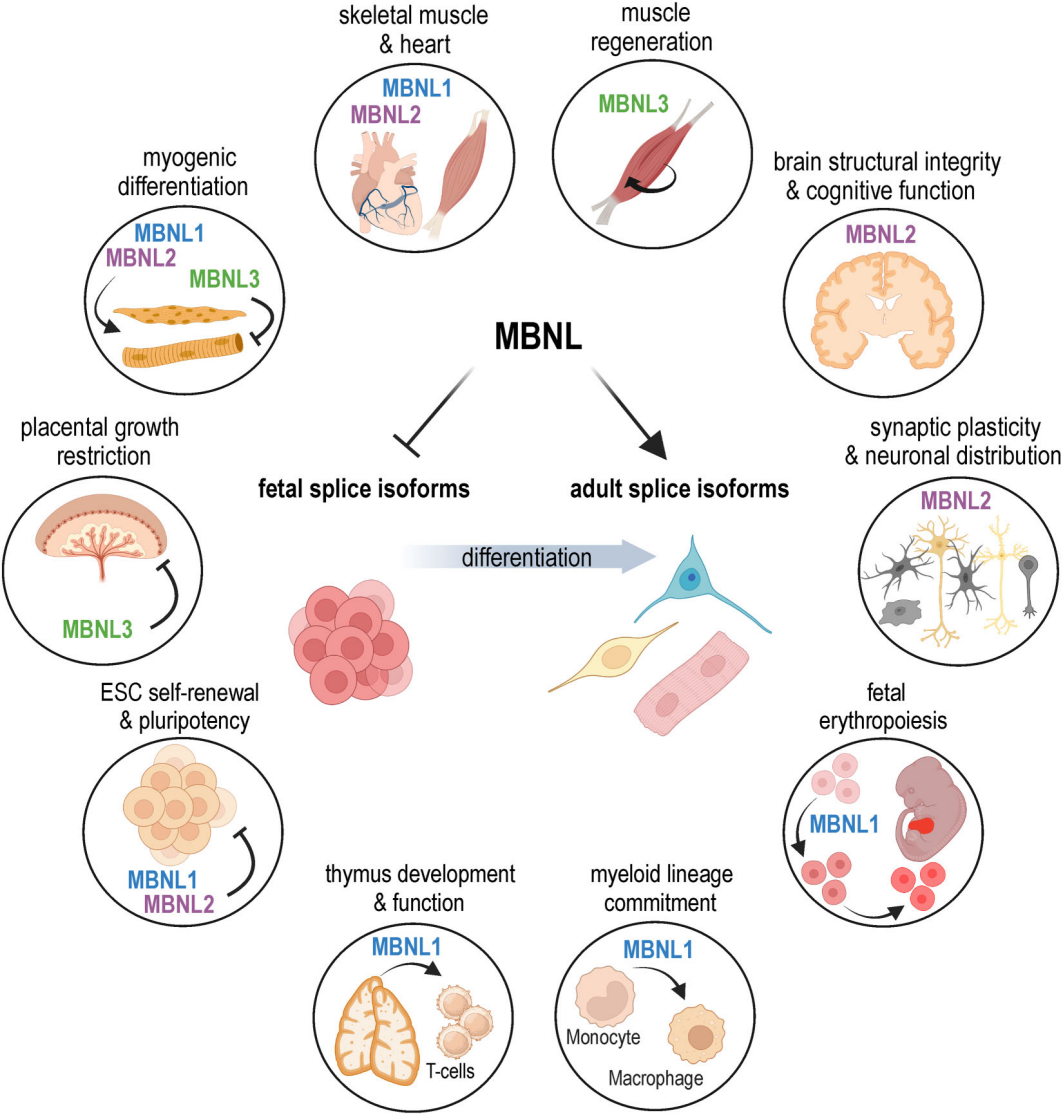
Main physiological roles of MBNL proteins. MBNLs are critically involved in the differentiation and maintenance of many tissues and organs, including muscle, the nervous system, the brain, and the hematopoietic and the immune systems. The specific roles of MBNL paralogs in these processes can be mutual or opposing, compensatory or redundant, as detailed in the main text. Figure created in BioRender (Stepniak-Konieczna, E. (2026), https://BioRender.com/kaumxto) and modified in CorelDraw.

As developmental sensors, MBNL proteins are vital for tissue differentiation and support diverse physiological processes, including cellular reprogramming, muscle and heart function, neural integrity and plasticity, and hematopoiesis (Fig. 5).

### Embryonic stem cell differentiation

MBNL1 and MBNL2 paralogs are direct negative regulators of a large program of cassette exon AS events that are characteristic of ESCs [96]. MBNL expression is minimal in ESCs compared with differentiated cell types, and in the latter, MBNL may repress ESC-differential exons and/or activate the inclusion of exons that are skipped in ESCs [96].

For example, conserved and partially redundant roles of MBNL1 and MBNL2 were demonstrated in the negative regulation of ESC-specific FOXP1/Foxp1 exon 18b/16b inclusion in humans and mice, respectively. The presence of this exon determines ESC self-renewal and pluripotency because it affects the DNA binding properties of the FOXP1 TF to promote the expression of core pluripotency genes, including *OCT4, NANOG*, and *SOX2* [96, 97]. Similarly, the OE of MBNL1 in ESCs triggered the silencing of those pluripotency factors upon differentiation and promoted the expression of specific lineage markers representative of all three germ layers, whereas the knockdown of *MBNL1* in differentiated cells enhanced somatic cell reprogramming, leading to an ESC-like AS pattern [96].

Similarly, analyses of AS in skin fibroblasts, induced pluripotent stem cells (iPSCs) derived from these cells, and fibroblasts redifferentiated from iPSCs indicated that MBNL1 and RBFOX2 cooperatively establish a splicing program involved in pluripotent stem cell differentiation [98]. Consistent with these findings, knockdown of *MBNL1* mirrored the stem cell-specific AS pattern [96, 98].

The *in vivo* significance of MBNL-regulated AS in ESC biology was also demonstrated in a planarian flatworm model, where knockdown of MBNL or CELF orthologs revealed their direct antagonistic effects on planarian regeneration by affecting the stem cell-specific transcriptome [99]. Specifically, the negative regulation of stem cell characteristic AS by MBNLs was counteracted via the positive effect of CELF [99]. Thus, the functional interplay between MBNL and CELF may form an ancestral developmental regulatory switch module that controls the pluripotent versus differentiated transcriptome. Strikingly, these findings may have profound consequences for carcinogenesis, as cancer cells often possess a low MBNL expression profile that could facilitate reversion to an embryonic AS pattern and promote malignant reprogramming by increasing survival and self-renewal.

### Placenta development

Among the splicing factor genes differentially expressed between trophoblastic and embryonic mouse tissues, *Mbnl3* displays the strongest placenta-specific enrichment alongside very low levels of expression in most nonplacental tissues [2, 25]. *Mbnl2* is also expressed in the placenta, and both *Mbnl2* and *Mbnl3* coregulate AS and polyadenylation during placental development and embryo growth. However, whereas significant concomitant expression in most other tissues is preserved for *Mbnl2*, it is not observed for *Mbnl3* [25]. This is likely associated with the evolution of a novel promoter and the emergence of a short isoform lacking the first ZnF pair [25] as well as the genomic localization of *Mbnl3* on the X chromosome.

MBNL3 restricts placental growth during mouse development via an inhibitory effect on cell proliferation through a *Myc*-dependent pathway [25]. RNA-seq analyses of *Mbnl3* KO placentas revealed the upregulation of *Myc* and its associated regulatory network, which is consistent with an increase in proliferation contributing to the larger placentas observed in mutant mice. The association between MBNL3 and Myc seems to be indirect, as no evidence for MBNL3 binding directly to *Myc* transcripts has been reported thus far [7, 25, 53].

### Skeletal muscle biology

MBNL proteins are required for myogenic differentiation throughout development and normal muscle function in adults, including postnatal muscle remodeling. The overall increase in MBNL proteins during muscle differentiation coincides with the fetal-to-adult splicing transition of hundreds of target RNAs [44].

The expression of individual *MBNL* genes and the specific functions of MBNL proteins differ greatly during these processes [100]. While *MBNL1* governs the majority of functions in developing and adult skeletal muscle [2, 100], *MBNL2* is downregulated during myogenic differentiation [100, 101] and remains at low levels in adult muscle tissue [6]. Specifically, direct comparison experiments revealed a marked increase in *MBNL1* expression during muscle differentiation, with an approximately 10-fold lower level of *MBNL2* and hardly any *MBNL3* in the adult muscle, and transient *MBNL3* activation during the early stages of regeneration [2, 3, 6, 23, 100–102]. The dominance of the MBNL1 paralog in the mature striated muscle explains why ablation of the mouse *Mbnl1* gene alone is sufficient to cause some muscular DM features [19], whereas mice deficient in *Mbnl2* fail to show an obvious muscular phenotype [6].

During postnatal skeletal muscle development, MBNL1 and MBNL2 regulate, in addition to mRNA splicing, microtranscriptome dynamics by influencing miRNA biogenesis through the AS of primary miRNA transcripts [103]. Notably, MBNL1 seems to have a greater effect on miRNA biogenesis than MBNL2 does, which is consistent with its predominant splicing activity in skeletal muscle tissue. These findings imply that MBNL sequestration in DM may be partially responsible for the altered miRNA activity observed in patients’ tissues.

In stark contrast to the other paralogs, MBNL3 acts as an inhibitor of myogenic differentiation [7, 18]. MBNL3 is expressed mainly during embryonic development, whereas its levels decrease in muscle cells undergoing differentiation. Consistently, MBNL3 levels are low in adult cardiac and skeletal muscle [104]. The antagonistic effect of MBNL3 on muscle differentiation is exerted via multiple mechanisms, such as (i) the suppression of the early differentiation marker myogenin and the inhibition of sarcomeric myosin heavy chain (*MyHC*) induction [18], (ii) the inhibition of myogenic differentiation 1 gene (*MyoD*)-dependent myogenic gene transcription [8], and (iii) the repression of the alternatively spliced β-exon of myocyte enhancer factor 2D (*Mef2D)* to suppress its expression [105] while simultaneously promoting myoblast proliferation [7, 18, 104].

However, MBNL3 is essential for normal adult muscle satellite cell activation and myoblast function as well as for adult muscle tissue remodeling [7]. Loss of *Mbnl3* delays injury-induced regeneration of adult muscle and impairs muscle function [7]. In contrast, the regenerative capacity and satellite cell numbers were not affected by the deletion of *Mbnl1* or *Mbnl2* alone [106], despite earlier reports showing that MBNL2 expression remains high in regenerating muscle fibers, suggesting its role in muscle tissue remodeling [107].

### Cardiac muscle biology

Like in skeletal muscle, MBNL1 is also the dominant paralog in the heart, both embryonic and adult [2, 108, 109], with markedly lower MBNL2 levels and very low MBNL3 amounts [2]. MBNL1 is also the major driver of fetal-to-adult AS transitions of cardiac transcripts during late fetal and postnatal heart development in mammals [108].

While *Mbnl1* KO mice presented heart conductive abnormalities and fibrosis [75], *Mbnl1*-deficient mice in which the *Mbnl2* gene was conditionally deleted in the heart displayed more severe pathogenesis and a spontaneous lethal cardiac phenotype, mirroring the phenotype observed in DM patients [78]. This severe cardiac pathology could be explained by splicing alterations in specific transcripts as well as changes in gene expression and the resulting protein synthesis [78, 110, 111]. A well-studied example includes the cardiac isoform of troponin encoded by the *TNNT2* gene, one of the major targets of MBNL1 in cardiac tissue that determines the calcium sensitivity of muscle fibers [57, 112]. Depletion of MBNL1 triggers *TNNT2* e5 inclusion, giving rise to a protein isoform conferring different calcium sensitivity to the myofilament, which ultimately negatively affects the contractile properties of cardiomyocytes and reduces myocardial function [57, 112].

In developing cardiomyocytes, MBNLs regulate the AS of genes involved in vesicular trafficking, mechanosensing, cardiomyofibril formation, and mitochondrial homeostasis [78]. For example, dual loss of *Mbnl1* and *Mbnl2* disrupted the splicing of *Nexn* and *Ttn* (involved in mechanosensing, myofibril elasticity, and structural integrity), *Plekhm2, Prune2*, and *Aplp2* (involved in vesicular/postendocytic trafficking) and *Mff* (involved in the mitophagy/endocytic pathway) [78].

Furthermore, mis-splicing of transcripts encoding ion channel proteins, including *Scn5a, Kcnip2, Ryr2*, and *Camk2d*, suggests a critical role for the MBNL1 and MBNL2 proteins in DM cardiac pathogenesis via control of ion channel trafficking [78]. Loss of both paralogs also affects gene expression, causing, e.g. downregulation of genes critical for mitochondrial respiratory chain biogenesis or upregulation of proteins crucial for calcium buffering [78]. In particular, increased amounts of *Casq1* mRNA, encoding a protein that regulates calcium release from the sarcoplasmic reticulum in skeletal muscle, may, in the absence of MBNLs, substantially affect excitation–contraction coupling and signaling pathways that are essential for maintaining normal cardiac function [78].

Studies with conditional *Mbnl1* knockout mice revealed that MBNL1 regulates the AS of a network of cardiac transcripts crucial for intra- and intercellular transport (e.g. *Atp2a1, Junctin, Cacna1s, Ryr2*), cell survival (e.g. *Capn3, Sirt2*), sarcomere and cytoskeleton organization (e.g. *Trim55, Mapt, Pdlim3, Sorbs1, Fhod1*), and structural components of the sarcomere (e.g. *Myom1, Tnnt2, Zasp*) [75]. Consistently, MBNL1 depletion resulted in the persistence of embryonic splice isoforms of these mRNAs, ultimately leading to the development of DM-like cardiac disease.

### Nervous system development and function

While both MBNL1 and 2 play important roles in AS regulation during the formation and maintenance of synaptic architecture, neuronal distribution, and dendritic morphology in the developing brain [113], MBNL2 is the most predominantly expressed paralog in brain tissue, and its loss leads to widespread splicing abnormalities [6, 44].

MBNL2 regulates the AS of genes essential for neuronal function, including *Nmdar1*, a core component of the synaptic machinery involved in long-term potentiation, learning, and memory [6, 114]. It also controls neuronal survival, differentiation, and growth through AS of *Bdnf* and modulates dopaminergic signaling by splicing of the dopamine receptor D2 (*Drd2*) gene [115]. Beyond this, MBNL2 promotes adult splice isoforms during dendritic spine development through the AS of *Add1*, a key regulator of spinogenesis [116], and regulates splicing programs in the choroid plexus that influence ion homeostasis, secretory activity and cerebrospinal fluid composition [117].

Moreover, MBNL1 and MBNL2 jointly regulate brain structural integrity and development through coordinated control of AS, including the *MAPT* (Tau) transcript, and influence neuroanatomy and behavior [118–120]. MBNL1 additionally promotes neurite outgrowth and rescues neurite morphogenesis defects in DM [121], whereas MBNL2 uniquely regulates REM sleep and cognitive function [6]. Both paralogs also contribute to neuromuscular junction maintenance and motor neuron neurotransmission by regulating the splicing of multiple genes involved in synaptic vesicle homeostasis, neurotransmitter release, and RNA localization [122].

Finally, impaired MBNL function has been linked to neurodevelopmental disorders, including autism spectrum disorders, as MBNL loss in mouse models induces developmental mis-splicing of autism-risk genes accompanied by social behavioral deficits and altered responses to novelty [123].

### Hematopoiesis and the immune system

During mammalian development, the fetal liver constitutes an important center of hematopoiesis, a layered and multistep process of commitment to erythrocytes and lymphoid as well as myeloid lineage cells. Although the loss of neither MBNL paralog is associated with developmental liver defects, MBNL1 plays essential roles in fetal erythropoiesis [124]. *Mbnl1* expression was detected in both fetal and adult stages of erythropoiesis, whereas *Mbnl2* was detected only in the adult stage and *Mbnl3* in neither erythropoietic stage (The Erythron Database; [125] and [124]). In contrast, human fetal and liver samples presented significant levels of all *MBNL* mRNA paralogs [2]. The knockdown of *Mbnl1* in cultured murine fetal liver cells impaired terminal proliferation and differentiation of erythroid progenitors by disrupting developmentally regulated exon skipping in the *Ndel1* transcript [124].

Conversely, another study demonstrated only a modest effect of *Mbnl1* loss on hematopoiesis under both steady-state and distinct stress conditions [126]. In stark contrast, loss of MBNL1 significantly impaired the development and propagation of murine and human mixed lineage leukemia (MLL)-rearranged leukemia *in vitro* and *in vivo* [126]. These findings indicate that although MBNL1 is dispensable for steady-state murine hematopoiesis, it is essential for the survival and propagation of transformed hematopoietic cells.

*Mbnl1* is highly expressed in the human [2] and murine thymus [14] and promotes its development and function [127]. Loss of *Mbnl1* led to aberrant thymic gene expression, pre-mRNA misprocessing, and mis-splicing of factors regulating thymocyte/T-cell development, including TFs of the TCF/Lef family (e.g. *LEF1* e6). These events ultimately lead to postnatal thymic hyperplasia with thymocyte accumulation, highlighting the role of MBNL1 in the immune system [127].

High-depth RNA-seq profiling of human primary monocytes differentiated into macrophages revealed that MBNL1 is a critical regulator of myeloid lineage commitment [128]. In contrast to the general trend of increasing MBNL1 levels during differentiation, MBNL1 is relatively abundant in monocytes but declines as they differentiate into macrophages, and many differentiation-associated splicing changes overlap known MBNL1 targets. Interestingly, knockdown of *MBNL1* in primary monocytes recapitulated several AS changes observed in differentiating monocytes, but their differentiation into macrophages was in fact impaired [128]. Although this seems paradoxical, it implies that not only the presence of MBNL1, but also timing, context as well as coordination with other factors may be crucial for the proper acquisition of macrophage identity and function. In summary, MBNL1 deficiency may have pronounced physiological consequences for processes involving inflammation and wound healing, especially in immunodeficiencies, autoimmune diseases, atherosclerosis, but also myotonic dystrophy and cancer.

## Multifaceted roles of MBNLs in pathological conditions

### MBNL dysfunction in myotonic dystrophy

Myotonic dystrophy, a highly variable multisystem disorder manifested mainly by muscle hyperexcitability (myotonia) and overall muscular weakness and atrophy, is the most common form of muscular dystrophy in adulthood. DM patients also suffer from heart conductance disturbances, arrhythmia, dilated cardiomyopathy, and heart failure, with males generally exhibiting a more severe phenotype than affected women do [129]. Common features include insulin-resistance and cataracts [15], whereas postmortem studies revealed fibrosis and fatty infiltration in patients’ hearts [130].

### Genetic background of DM

The two genetically distinct types of DM, DM type 1 (DM1; OMIM #160900) and DM type 2 (DM2; OMIM #602668), are caused by nucleotide repeat expansion within noncoding regions of two unrelated genes: a (CTG)n triplet expansion within the 3′UTR of the dystrophia myotonica protein kinase (*DMPK*) gene [15–17] and a (CCTG)n tetranucleotide repeat expansion within intron 1 of the cellular nucleic acid binding protein (*CNBP*) gene [131–136], respectively. Although both genes are protein-coding genes, the primary pathogenic mechanism of DM1 and DM2 does not involve deficiency of the protein products, but rather RNA gain-of-function toxicity and multiple downstream events, including MBNL sequestration.

The size of DM1 expansion strongly correlates with disease severity, likely due to the efficiency of MBNL sequestration on expanded RNA derived from the mutated gene [3], whereas no such clear correlation was demonstrated for DM2 [137]. The DM expansion size is highly variable due to germline variation that affects intergenerational differences, as well as substantial somatic expansion and mosaicism [15, 138–141]. In DM1, notably, the skeletal muscle and the heart in particular show greater CTG expansion than other tissues, e.g. blood [142]. Apart from genetic background, the two types of DM differ in onset and phenotype, with DM1 being more severe, presenting earlier, and predominantly affecting distal muscles, whereas DM2 is milder and commonly involves proximal muscles.

### Toxic RNA, foci and MBNL sequestration in DM

Mutant transcripts carrying pathogenic C/CUG expansions (C/CUG^exp^) assemble thermodynamically stable double-stranded RNA hairpin structures that are retained in the nucleus as discrete aggregates, termed foci or ribonuclear inclusions, that attract and sequester distinct poly(CUG)-binding RBPs, primarily MBNLs [143–148]. Nuclear RNA foci were first described more than three decades ago as aberrant membraneless structures [149]. However, their composition, modulators and assembly mechanisms are still not well understood. Recently, intermolecular interactions of repeat-expanded RNAs were proposed to lead to their phase separation into gel-like condensates *in vitro* and in transfected cells overexpressing CAG expanded RNA [150]. Yet, this does not fully align with studies showing very low per-cell expression of mutant *DMPK* transcripts, suggesting that DM1 foci comprise only one or a few mutant RNAs [151, 152]. Accordingly, a very recent study in a DM1 patient-derived myotube model demonstrated that the majority of CUG^exp^ RNA foci contain single RNA species and that rare multimeric foci are associated with transcription sites, as evidenced by quantitative single-RNA imaging [153].

Early findings that all three mammalian MBNL paralogs colocalize with nuclear foci of CUG^exp^/CCUG^exp^ RNA [3, 4, 102] shaped the initial hypothesis of a direct link between MBNL deficiency and a pathogenic process and provided the first glimpse into the molecular mechanism underlying DM. The exact role of MBNL proteins in repeat RNA foci formation is not fully understood, but several findings imply their direct role in facilitating foci assembly. MBNLs may stabilize foci through dimerization [35] and potentially through multivalent and simultaneous binding of distinct RNA species via four ZnFs [24]. This “crosslinking” capacity of MBNLs has been further supported by decreased numbers of multimeric RNA foci in DM1 myoblasts with a knockdown of *MBNL1* and *MBNL2* [153].

MBNL proteins are not merely trapped in foci, but assemble labile nuclear aggregates with constantly forming and disaggregating structures, causing free versus sequestered pools of MBNL to be very dynamic [49, 154]. Data indicate that when CUG^exp^ foci are saturated with MBNLs (e.g. when expansions are relatively small), the proteins may freely dissociate from these structures and are rapidly exchanged by nucleoplasmic MBNL proteins. Conversely, when unoccupied binding sites are still available within longer expansions, MBNL proteins circulate within RNA foci, continuously changing the intrafoci binding sites on CUG^exp^ transcripts [49]. The latter, unsaturated model, takes into account the stoichiometry of MBNL and its binding sites, thus partly explaining the mechanism underlying increasing sequestration and splicing deregulation as the disease progresses [49].

**Figure 6.**
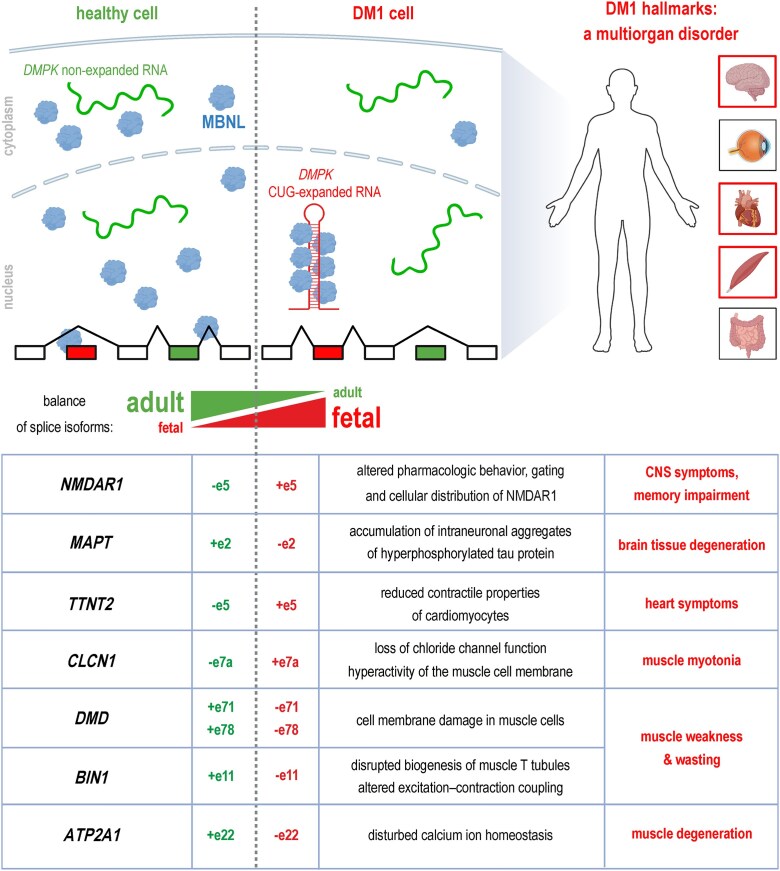
Molecular hallmarks of DM1. The hallmarks of DM1 largely result from the insufficiency of MBNL proteins and the ensuing imbalance of AS. In healthy cells, non-expanded *DMPK* RNAs are present in the cytoplasmic and nuclear compartments and do not interfere with the ability of MBNLs to promote adult splice isoforms. In DM1 cells, mutant *DMPK* RNAs derived from the CTG^exp^ allele are predominantly present in the nuclear compartment, where they attract, bind and trap MBNLs within ribonuclear foci. Ultimately, functional depletion of MBNLs alters the splicing pattern, leading to an imbalance of adult and fetal-like mRNA isoforms. Predominant fetal-like transcripts give rise to proteins unable to function properly in adult tissues, leading to multiple pathologies in distinct organs of DM patients, including the brain, eyes, skeletal muscle, heart, and gastrointestinal tract. Examples of aberrantly spliced transcripts contributing to DM1 hallmarks in selected tissues are illustrated and discussed in the main text. Figure created in BioRender (Stepniak-Konieczna, E. (2026) https://BioRender.com/r4mscbl) and modified in CorelDraw.

The repeat-imposed sequestration depletes the cellular pool of active MBNLs, causing their functional imbalance and consequently hampering the AS of hundreds of target pre-mRNAs. As a result, a shift in the splicing pattern occurs, mimicking the pattern observed in fetal and neonatal tissues (DM spliceopathy) (Fig. 6). Cumulatively, these splicing alterations lead to production of protein isoforms unable to perform their functions in adults, ultimately driving pathological changes in multiple tissues, particularly muscle.

### MBNL-dependent spliceopathy in DM

While a detailed spectrum of AS alterations in the adult DM1 muscle has been reviewed extensively [155, 156], we highlight selected MBNL-dependent AS events that are disrupted in muscle and cause major DM1 hallmarks, such as myotonia, muscle weakness, and wasting (Fig. 6).

One of the best studied MBNL1 targets in muscle is chloride voltage-gated channel 1 (*CLCN1*), which encodes a chloride channel essential for normal muscle contraction and relaxation. Reversal to the fetal splice pattern of *CLCN1* in MBNL-depleted DM1 skeletal muscle causes a failure to produce the adult isoform of the CLCN1 protein, leading to prolonged muscle contractions and delayed relaxation due to defective ion conductance and excitability, defined as myotonia [157, 158]. Specifically, MBNL1 exhaustion triggers an in-frame inclusion of intron 2 and exon 7a of *CLCN1*, generating a premature stop codon. The truncated CLCN1 protein cannot localize within the membrane, leading to its hyperexcitability [108, 159–161].

Similarly, a proper calcium-dependent contraction/relaxation cycle within the muscle depends on MBNL1-regulated AS of three main transcripts encoding the calcium channel CaV1.1 (CACNA1S), ryanodine receptor 1 (RYR1) and sarcoplasmic/endoplasmic reticulum Ca^2+^-ATPase SERCA1 (ATP2A1) [162, 163]. Depletion of MBNL1 in DM1-muscles promotes neonatal splicing isoforms of these transcripts, such that they lack e29, e70, or e22, respectively, thus contributing to muscle weakness via altered excitation–contraction coupling (*CACNA1S*), decreased muscle contraction (*RYR1)* and muscle degeneration owing to impaired intracellular calcium homeostasis *(ATP2A1)*.

Furthermore, proper tubular invaginations of muscle membranes and the biogenesis of muscle T tubules are regulated via MBNL1-dependent AS of bridging integrator-1 (*BIN1*). Loss of functional MBNL1 in DM promotes *BIN1* isoforms lacking exon 11 and the generation of an inactive form of the BIN1 protein, leading to muscle weakness due to altered excitation–contraction coupling [164]. Similarly, increased exclusion of dystrophin (*DMD*) exons 71 and 78 [165] in the absence of MBNL1 compromises muscle membrane integrity, leading to muscle weakness [166, 167].

### Dysfunction of MBNLs in other nucleotide repeat-expansion disorders

The pathogenicity of aberrant MBNL localization and function has been implicated in a subset of rare neurodegenerative diseases caused by exonic CAG trinucleotide repeat expansions that are translated into abnormally long polyglutamine (polyQ) tracts, including Huntington’s disease (HD, [168]), HD-like 2 (HDL2, [169]), and spinocerebellar ataxia type 3 (SCA3, [170]) and 8 (SCA8, [171]). Other tandem repeat disorders associated with MBNL aberrations include fragile X-associated tremor/ataxia syndrome (FXTAS, [172]), fragile X syndrome (FXS, [173]), and amyotrophic lateral sclerosis (ALS, [174]).

CUG-expanded RNAs have been detected in polyQ diseases [175–177]. For example, in HDL2, bidirectional transcription from the *JPH3* locus generates CAG^exp^ RNAs [178], as well as CUG^exp^ repeats containing mutant *JPH3* RNAs that bind MBNL1, a process linked to disrupted *MAPT* and *APP* splicing in the brain cortex, albeit to a lesser degree than in DM [177]. Similarly, in SCA8, bidirectional transcription generates two transcripts with expanded repeats [171, 179]. The CAG^exp^  *ATXN8* RNA is translated in different frames and generates, among others, polyQ proteins [176, 180, 181], whereas the untranslated CUG^exp^  *ATXN8OS* transcript accumulates in the nucleus, sequestering MBNL [182].

A genetic screen in *Drosophila* overexpressing SCA8 with a CTG expansion identified *muscleblind* as one of the key dominant modifiers and enhancers of SCA8-induced late-onset progressive neurodegeneration in the retina [183]. Similarly, an OE-based screen in a transgenic *Drosophila* SCA3 model revealed an insertional mutation in the promoter of the *mbl* gene that upregulated *mbl*, markedly enhancing polyQ-induced eye degeneration [170]. Increased Mbl elevated both polyQ RNA and protein levels, accelerating inclusion formation, and this effect was conserved in transgenic flies coexpressing pathogenic SCA3 with the human major isoform 40 of MBNL1 [170]. Notably, the human MBNL1 isoform lacking exon 4, which is critical for optimal CUG repeat binding and conserved in *Drosophila* Mbl, showed only mild enhancement, indicating that MBNL1 modulates polyQ toxicity primarily through RNA-dependent mechanisms [170]. Interestingly, not only CUG^exp^ repeats but also CAG^exp^ RNAs were found to bind MBNL1 *in vitro* and *in vivo* in HD and SCA3 cells and may therefore contribute to the overall toxicity observed in polyQ diseases, among other processes, including repeat-associated non-AUG (RAN) translation of toxic proteins from these mutant transcripts [2, 170, 184].

Degeneration of neurons in FXTAS, a disorder caused by limited expansion of CGG trinucleotides in the 5′ untranslated region of the *FMR1* gene [185–191], could be partially attributed to sequestration of a number of proteins on the mutated *FMR1* mRNA, including MBNL1 [192–196], as well as toxicity of the polyglycine protein translated from the mutated mRNA upstream to the CGG repeats [197–201]. Although MBNL1 was detected in both ubiquitin-positive inclusions and CGG aggregates [192, 194], its splicing function remained largely unaltered in FXTAS patients, indicating that it was not immobilized in the FXTAS-associated inclusions and could still perform its functions [192]. The situation appears to be different in FXS, in which greater expansion of the CGG repeat completely abrogates translation of the FMRP protein. Specifically, *Fmr1*-deficient mice exhibited widespread mis-splicing in all brain regions and peripheral tissues of the mutant animals, which were in major part contributed to the incresed activity of MBNL1 and MBNL2 [202]. The study indicated that in healthy tissue, *Mbnl1/2* transcripts are translationally inhibited by direct interaction with FMRP, and in the absence of the latter, an increase in *Mbnl1* and *Mbnl2* translation results in frequent autoregulatory skipping of NLS-containing exon 5 from their pre-mRNAs. This, in turn, alters the MBNL nucleus-cytoplasm distribution and affects the splicing decisions on target mRNAs, contributing to the overall pathology [202].

Finally, MBNL proteins affect FUS (fused in sarcoma)-associated ALS, a neurodegenerative disease marked by degeneration of motor neurons and progressive denervation of voluntary muscles [174, 203]. FUS is a predominantly nuclear DNA- and RNA-interacting protein [204] that is essential for multiple steps of RNA processing [205]. In ALS, mutant FUS accumulates in stress granules, disrupting their dynamics and causing RNA processing dysregulation and cellular toxicity [206, 207]. Notably, MBNL1 knockdown in FUS-mutant neurons rescues dendritic defects and cellular toxicity by indirectly releasing FUS from stress granules via the restoration of survival motor neuron (SMN) protein localization [206]. In contrast, MBNL depletion does not suppress the toxicity associated with other ALS-linked proteins, such as TDP-43 [206, 208]. Taken togehter, these findings indicate that MBNL modulation confers a therapeutic benefit specifically in FUS-associated ALS.

### Dual roles of MBNLs in cancer

**Figure 7.**
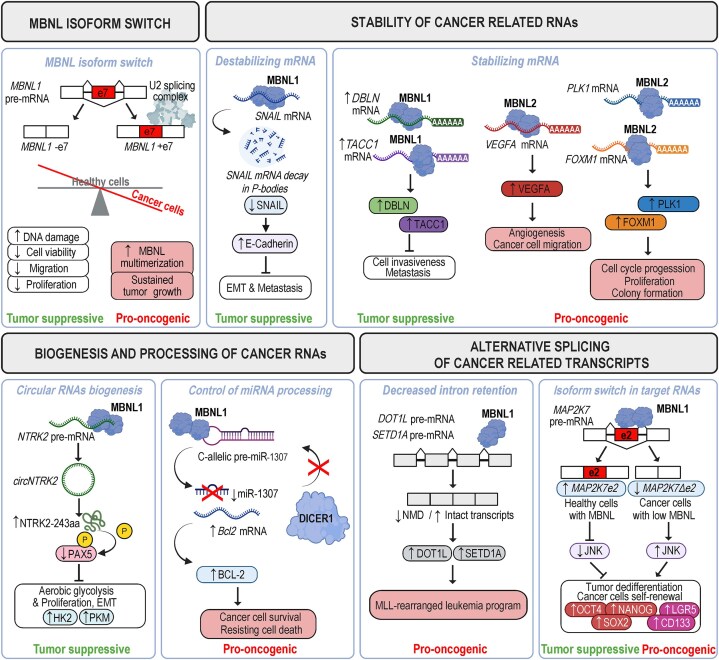
Dual roles of MBNLs in cancer. MBNLs may affect virtually all cancer hallmarks through several basic molecular mechanisms involving isoform switching [209]; RNA biogenesis, processing, and stability [216–218, 221, 222]; and splicing control of cancer-related transcript networks [126, 224]. Figure created in BioRender (Stepniak-Konieczna, E. (2026) https://BioRender.com/xj17wk3) and modified in CorelDraw.

Pervasive dysregulation of the transcriptome in cancer implicates MBNLs in tumorigenesis, with emerging evidence revealing their context-dependent and dual roles—as tumor suppressors or oncogenic drivers. The selected examples illustrate that these functions arise through several core mechanisms—including AS of cancer-related RNAs, regulation of transcript stability, and control of RNA biogenesis and processing—often influenced by MBNL isoform switching. Through these mechanisms, MBNLs can modulate virtually all cancer hallmarks, from cell cycle control and survival signaling to angiogenesis, invasion, and metastasis (Fig. 7).

### Dysregulation of MBNLs in cancer

Multiple factors contribute to the frequent overall downregulation of *MBNL1* and *MBNL2* expression in diverse cancers, which is correlated with poor prognosis ([209–211] and recently reviewed in [54]). These include, e.g. epigenetic mechanisms such as promoter hypermethylation or microRNA-based suppression, as shown in the case of *MBNL2* downregulation in breast, lung, and liver cancer [212], or the tumor microenvironment itself, particularly hypoxia, which may reduce the functional output of the protein by altering its subcellular localization. The latter was shown for MBNL1, whose nuclear export under hypoxia reduced its activity in glioblastoma (GBM) [213].

However, some tumors exhibit a paradoxical upregulation of MBNLs. In hepatocellular carcinomas (HCCs), upregulation of MBNL2 has protective effects, as its knockdown triggers a more aggressive phenotype through supporting cancer cell proliferation and migration [214]. Similarly, *MBNL3* expression is activated in HCC by the stem cell TFs NANOG, OCT4, and SOX2 [215] and is generally associated with poor prognosis and therapy resistance. These differences highlight the context-dependent roles of MBNLs in tumorigenesis.

### MBNL isoform switching in cancer

A simple isoform switch may control the tumor-suppressive vs. pro-oncogenic roles of MBNLs. For example, e7 of *MBNL1*, which is required for protein homodimerization, is the most differentially included exon in cancer, despite overall *MBNL1* downregulation in tumor samples [209]. Exon 7-deficient MBNL1 functions as an anti-survival, tumor-suppressive factor that cancer cells selectively downregulate in favor of e7-containing isoforms (Fig. 7). This is because e7-depleted isoforms give rise to dominant-negative proteins that disrupt the expression and/or splicing of genes controlling proliferation, cell cycle checkpoints, and chromosome segregation, leading to DNA damage and reduced cancer cell survival and migration [209]. In contrast, e7-included MBNL1 isoforms, promoted by U2 splicing complex components, enhance cancer cell survival and migration, particularly in prostate cancer [209].

Inactivation of MBNL1 splicing activity via isoform switching in aggressive brain cancer GBM is yet another example. Here, the hypoxic tumor microenvironment promotes a shift from e5-containing to e5-lacking *MBNL1* mRNAs, enhancing rapid nuclear export of the MBNL1 protein and facilitating the expression of numerous gene isoforms associated with an embryonic stem cell-like state [213]. Overall, the MBNL isoform switch may determine the abundance and splicing of a key network of genes involved in cell migration, DNA repair, and cell division during cancer development.

### MBNL-controlled transcript stability in cancer

The role of MBNL in RNA processing and stability impacts hallmark cancer processes, including metastasis (Fig. 7). In colorectal cancer (CRC), MBNL1 suppresses tumorigenesis by binding and destabilizing *SNAIL* mRNA via its enhanced recruitment to P-bodies, leading to restored E-cadherin expression and inhibition of epithelial–mesenchymal transition (EMT) [216]. Conversely, in breast cancer, MBNL1 stabilizes transcripts implicated as metastasis suppressors, *DBLN* and *TACC1*, by directly binding to their 3′UTRs [217]. Consequently, upregulation of MBNL1 in both types of cancer is associated with reduced invasiveness and metastasis, whereas its downregulation is associated with increased cancer progression, metastasis, and poor prognosis [216, 217].

MBNL2, in contrast, increases tumorigenicity and appears to be an essential posttranscriptional regulator of the clear cell renal cell carcinoma transcriptome by enhancing the stability and abundance of transcripts in the HIF-α, FOXM1, and PLK1 pathways, which promote cell cycle progression, proliferation, and colony formation [218] (Fig. 7). For example, MBNL2 binds the 3′UTR of *VEGFA* mRNA, stabilizing it and increasing the protein level of VEGFA, a key angiogenic factor in the HIF-α–mediated hypoxic response [218]. Consistent with this role, MBNL2 is upregulated under hypoxia and supports tumor adaptation by sustaining the expression of hypoxia-responsive genes such as *VEGFA* [219].

### MBNL-mediated RNA biogenesis and processing in cancer

MBNLs affect tumorigenesis through their role in RNA biogenesis (Fig. 7). This is exemplified in esophageal squamous cell carcinoma, where downregulation of MBNL1 causes extensive downstream alterations in the expression of numerous circRNAs, lncRNAs, and mRNAs associated with tumor progression [220]. Similarly, a reduction in nuclear MBNL1 in GBM promotes aerobic glycolysis, a hallmark process of tumor development and malignancy [221]. Mechanistically, MBNL1 exerts antitumor activity in GBM by directly binding *NTRK2* pre-mRNA in the nucleus to promote its circularization (circNTRK2) and expression of the encoded NTRK2-243aa, which in turn reduces the half-life of PAX5 via phosphorylation, ultimately inhibiting the expression of PAX5-dependent glycolysis-related genes [221].

Conversely, MBNL1 promotes CRC initiation by directly binding the ‘UGCUGC’ motif in the stem loop of a C-allelic pre-miR-1307, prevalent in CRC patients, thus antagonizing its processing by DICER1 and ultimately leading to a lower level of miR-1307. This in turn elevates the expression of its direct target, the prosurvival and antiapoptotic protein BCL2, which protects cancer cells [222] (Fig. 7).

### MBNL-mediated AS in cancer

Compared with other leukemia types, *MBNL1* is one of the most consistently overexpressed genes in MLL [126]. ChIP-seq data revealed direct stimulation of the *MBNL1* promoter by the MLL fusion protein. Excessive MBNL1 directly interacts with and stabilizes transcripts of multiple leukemogenic genes (mostly through decreased intron retention). These include the histone methyltransferases *DOT1L* and *SETD1A*, which subsequently support the transcriptional activation of downstream targets of the MLL-fusion protein, including the activation of the *MBNL1* promoter itself [126]. This positive feedback loop ultimately alters the AS of target RNA transcripts, promoting cancer cell survival. Overall, these findings show that MBNL1-mediated RNA splicing is causal to the pathogenesis of MLL-rearranged leukemias.

Consistent with reports on the MBNL-mediated repression of the stem cell-specific AS program and promotion of stem cell differentiation [96, 98], MBNL1-low cancers and embryonic stem cells share a discrete set of common AS events [96, 223, 224]. A fair amount of data indicate that MBNLs promote AS events that suppress tumor dedifferentiation, and their loss or downregulation correlates with an increased stemness phenotype of cancer cells [96, 223, 224] (Fig. 7). One example involves MBNL1-promoted inclusion of e2 within *MAP2K7* pre-mRNA in healthy cells, whereas low MBNL1 levels in cancer allow its skipping. The resulting *MAP2K7∆* e2 splice variants increase the stem- and progenitor-like properties of cancer cells via c-Jun N-terminal kinase (JNK) activation and signaling [224]. Consistently, the increased tumorigenic properties of normal and cancer cells with MBNL1 knockdown were accompanied by the upregulation of cancer stem cell markers (*CD133* and *LGR5*) and pluripotency markers (*OCT4, NANOG*, and *SOX2*) [224]. In summary, understanding these functions is essential for developing MBNL-targeted, individualized therapeutic strategies.

## MBNLs as therapeutic targets in myotonic dystrophy

**Figure 8.**
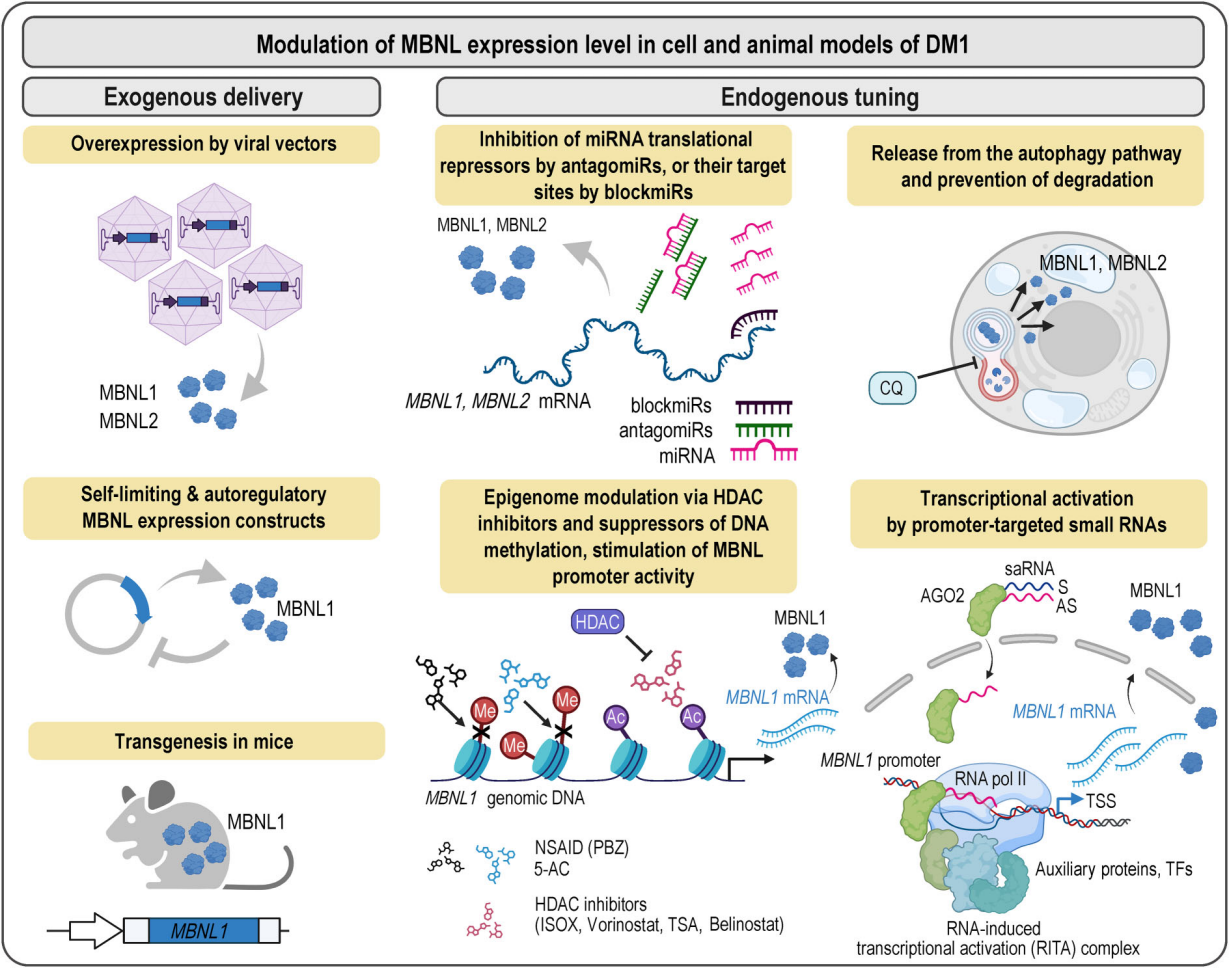
Selected MBNL-targeted approaches to alleviate DM1 pathogenesis. Figure created in BioRender (Stepniak-Konieczna, E. (2026) https://BioRender.com/ykj65so) and modified in CorelDraw.

Apart from symptomatic treatment and supportive care, there is no cure to fundamentally improve the course of DM. Distinct types of therapeutic strategies are being extensively tested with the aim of (i) removing the prime culprit, i.e. the expanded repeats, by repeat contraction, transcription blocking, or DNA editing (reviewed in [225, 226]), (ii) alleviating disease symptoms by either removing the toxic RNA (e.g. by antisense reagent-mediated degradation) or inhibiting its interaction with MBNL proteins to prevent their sequestration (e.g. by small-molecule compounds or antisense reagents; reviewed in [226, 227]), or (iii) modulating *MBNL* expression to restore the pool of functional proteins. Here, we outline selected MBNL-directed approaches grounded either in the OE of various exogenous MBNL isoforms or in the modulation of the endogenous *MBNL1*, aiming to increase the protein amount and counteract the effect of toxic RNA-imposed sequestration (Fig. 8 and Table 4).

**Table 4.** Selected modulators of the endogenous MBNL expression

Name	Compound type	Effect on endogenous MBNL/MOA	References
ISOX	Generic HDAC inhibitor	∼1.5-fold increase in MBNL1 protein in DM1 fibroblasts;**MOA:** unknown	[239]
Vorinostat	Pan-HDAC inhibitor	∼2-fold [239] and up to ∼1.5-fold [240] increase in MBNL1 protein in DM1 fibroblasts and myoblasts, respectively;**MOA:** presumably post-transcriptional, precise mechanism unknown	[239, 240]
Phenylbutazone	NSAID	∼1.3–1.9-fold increase in *Mbnl1* mRNA and protein expression in C2C12 cells; ∼2–6-fold increase in *Mbnl1* mRNA level in *HSA*^LR^ mice;**MOA:** suppression of methylation of MeR2 region in *Mbnl1* DNA	[26]
5-aza(deoxy)cytidine	Global DNA methylation inhibitor	∼2-fold *Mbnl1* mRNA upregulation in C2C12 cells;**MOA:** presumably suppression of DNA methylation	[26]
Ketoprofen	NSAID	∼1.2-fold increase in *Mbnl1* mRNA level in C2C12 cells;**MOA:** unknown	[26]
Calcitriol	Active form of vitamin D3	∼2-fold increase in *Mbnl1* mRNA and protein expression in C2C12 cells and *HSA*^LR^ mouse model;**MOA:** activates *Mbnl1* promoter, precise mechanism unknown	[243]
Furamidine	Small molecule binding CTG/CAG repeat DNA	∼2-fold increase in *MBNL1/2* transcript and protein;**MOA:** unknown, speculations include interactions with DNA in *MBNL1/2* genes, altered TF binding or interaction directly with *MBNL1/2* transcripts and their stabilization	[245]
Chloroquine (CQ)	Autophagy blocker	∼3-fold increase in *Mbl* mRNA and protein expression in a *Drosophila* model; ∼2-fold increase in MBNL1 protein expression in patient-derived myoblasts;**MOA:** blocks autophagosome fusion with lysosomes and promotes MBNL release from the autophagic pathway hyperactivated in DM, thus preventing its degradation	[246]
AntagomiRs / antimiRs	Antisense oligomer	∼1.5–4-fold increase in *MBNL1/2* mRNA and protein expression in DM1 cells and HSA^LR^ mouse model;**MOA:** antisense blocking of miRNA translational inhibitors of MBNL1 (miR-23b and miR-218)	[30–32, 249]
BlockmiRs	Antisense oligomer	∼1.5–4-fold increase in *MBNL1/2* mRNA and protein expression in DM1 cells and HSA^LR^ mouse model;**MOA:** antisense blocking of miR-23b and miR-218 target binding sites within *MBNL1* 3′UTR	[33, 250]
saMB1_1 and saMB1_2	saRNA duplexes	∼2–3-fold increase in *MBNL1* mRNA and protein level in DM1 cells;**MOA:** direct on-site effect of saRNA on *MBNL1* promoter – AGO2-mediated loading of the antisense strand onto target sequence, recruitment of RITA components and auxiliary proteins stimulating transcription	[251]

Abbreviations: HDAC, histone deacetylase; NSAID, nonsteroidal anti-inflammatory drug; saRNA, small activating RNA; MOA, mechanism of action.

### *MBNL* gene replacement strategies

A seminal study in *HSA*^LR^ DM1 mouse model [228], which used intramuscular delivery of adeno-associated viral vector serotype 2 (AAV2) carrying a single *Mbnl1/41* isoform, revealed that even a modest increase in MBNL1 can alleviate DM1 symptoms [229]. Although relatively low levels of protein upregulation were achieved (only a 2-fold increase), they sufficiently rescued myotonia and mis-splicing of several AS events in *HSA*^LR^ muscles, including the chloride channel *Clcn1* e7a underlying myotonia [157, 159–161]. However, the normal myofiber structure was not restored, possibly because the *Mbnl1* 41-kDa isoform, which is expressed mainly in neonatal but not adult skeletal muscle, was insufficient to fully rescue the DM1 phenotype.

In support of this, constitutive multisystemic OE of a humanized *MBNL1* 40-kDa transgene partially corrected spliceopathy, myotonia, and myopathy in the *HSA*^LR^ mouse model [230]. The expression levels of the recombinant protein in these transgenic mice were much greater than those achieved with viral transduction (up to 17-fold in comparison with 2-fold), which likely contributed to the premature fetal-to-adult AS transition of MBNL1 targets in skeletal muscle early in embryonic development. Even with this premature transition, the skeletal muscle developed and functioned normally, suggesting a compensatory mechanism allowing muscle development despite the expression of adult isoforms of MBNL1 target transcripts [230].

Intriguingly, AAV9-mediated delivery of the *Mbnl2* 40-kDa isoform in the *HSA*^LR^ model partially reversed the fetal splicing pattern of several DM1 biomarker exons, particularly in paraspinal muscles [231]. This implies that combined therapeutic strategies involving OE of both paralogs may be more effective, as MBNL1 and MBNL2 share some targets but also regulate distinct RNA subsets. This is corroborated by the much more severe DM1 phenotype in compound *Mbnl1* and *Mbnl2* mutant mice [73, 232].

Although delivery of *MBNL* coding sequences via AAV vectors could offer robust OE with therapeutic advantages, the delivery method itself has inherent drawbacks that should be taken into account in therapeutic designs [165, 233]. Furthermore, unopposed and uncontrolled expression of the protein may drive deleterious effects on its own. For example, OE of *MBNL1* in mouse embryonic fibroblasts led to myofibroblast transformation, and in mice, it elicited a progressive fibrotic response in multiple tissues and organs [234]. These data necessitate controlled and context-dependent *MBNL* OE in therapeutic strategies but also indicate that the therapeutic impact may depend strictly on the genetic context. For example, when transgenic mice overexpressing the *MBNL1* 40-kDa isoform [230] were crossed with DM200^+^ mice expressing the *DMPK* 3′UTR with ∼200 toxic CUG repeats as a part of an inducible RNA transcript encoding GFP [235], no therapeutic effect on myotonia or skeletal muscle function was observed [236]. Conversely, *MBNL1* OE in this context impaired cardiac function, whereas DM1 splicing abnormalities persisted regardless of *MBNL1* upregulation. The genetic context of the expanded CTG repeated region may be crucial, as a significant therapeutic effect of *MBNL1* OE was observed in the *HSA*^LR^ mice, where CTG repeats are incorporated in the 3′UTR of the human skeletal actin gene [229].

Finally, recent work with OE of truncated *MBNL1* containing both ZnF domains but lacking the C-terminal domain (MBNL1∆-decoy) demonstrated its potential as a therapeutic decoy for CUG^exp^ repeats, resulting in the release of endogenous MBNL1 from sequestration [237]. This decoy interferes with foci dynamics and competes with endogenous MBNL1 for CUG^exp^ binding. It also forms less stable aggregates with expanded repeats than MBNL1. Notably, AAV-delivered MBNL1∆-decoy had a prolonged therapeutic effect in the *HSA*^LR^ DM1 mouse model, reversing the spliceopathy in skeletal muscle and ameliorating disease pathology.

MBNL1 autoregulation is an appealing concept that could be explored in therapeutic designs to eliminate unwanted effects associated with protein excess and improve safety. This idea was tested via OE of hybrid constructs carrying the MBNL1 CDS, in which an MBNL-sensitive *ATP2A1* exon 22 (e22) containing an in-frame stop codon was inserted between e2 and e3, allowing MBNL-dependent e22 inclusion and consequent shutdown of protein production [238]. This approach enabled efficient correction of AS abnormalities in a cellular model of DM1, while at the same time preventing excess protein production.

### Modulation of endogenous *MBNL* expression

Whereas OE usually involves a single cDNA variant delivered with recombinant AAV vectors, potential endogenous modulation with small molecules or RNA oligonucleotides offers the advantage of correct stoichiometric expression to achieve balanced splice isoform diversity. We summarize key compounds, including pharmacological modulators of the epigenome as well as antisense-based reagents, that have been reported to increase endogenous MBNLs via distinct mechanisms sufficiently to trigger therapeutic benefits in DM1 cells and mouse models (Fig. 8 and Table 4).

### Epigenetic modifications of *MBNL* expression

Two small molecule histone deacetylase (HDAC) inhibitors, ISOX and vorinostat, increased endogenous *MBNL1* expression in DM1 fibroblasts 2-fold and significantly corrected the splicing of several MBNL1 target transcripts [239]. Accordingly, high-throughput screening of FDA-approved compounds indicated that vorinostat is a strong inhibitor of CUG foci formation in DM1 myoblasts [240]. In addition, it increased the MBNL1 protein level in a dose-dependent manner by up to 2-fold but simultaneously decreased the *MBNL1* mRNA level, suggesting a mechanism of action other than transcription stimulation, e.g. increased bioavailability of the MBNL1 protein due to its release from foci and/or reduced foci formation. Several other pan-HDAC inhibitors, including trichostatin A (TSA) and belinostat, similarly alleviated pathogenic DM1 features [240].

Furthermore, drug repositioning-based screening identified a nonsteroidal anti-inflammatory drug (NSAID) phenylbutazone (PBZ), which suppressed methylation at the methylated region MeR2 in the *Mbnl1* locus (in the intron preceding the first coding exon) and triggered the upregulation of *Mbnl1* transcription, indicating important enhancer activity within this genomic region. PBZ increased *Mbnl1* expression from 1.3-fold up to 1.9-fold in cultured cells (C2C12) and from 2-fold up to 6-fold in *HSA*^LR^ mice, resulting in a partial rescue of AS defects and decreased numbers of abnormal central nuclei in muscle fibers [26]. The suppressive effect of DNA methylation on *Mbnl1* expression was also demonstrated via the global suppressor of DNA methylation, 5-aza(deoxy)cytidine (5-AC), which increased *Mbnl1* mRNA levels by more than 2-fold in C2C12 cells [26].

While PBZ is a nonselective cyclooxygenase (COX) inhibitor, knockdown of the COX-1 isoform in C2C12 myoblasts and myotubes led to an ∼1.5-fold increase in the MBNL1 protein level [241]. In agreement, bisulfite sequencing confirmed that COX-1 knockdown, similar to PBZ treatment, suppressed the methylation of MeR2, indicating that COX-1-mediated pathway inhibition may be one of the key triggers of *Mbnl1* transcriptional regulation. Interestingly, another NSAID, ketoprofen, which was identified as a chemical modifier and suppressor of CUG-induced toxicity in a *Drosophila* model of DM [242], also upregulated the expression of *Mbnl1* in C2C12 cells, implying that NSAIDs may have a general beneficial effect on DM [26].

### Other small molecules affecting *MBNL* expression

A more recent study demonstrated that calcitriol, an active form of vitamin D, increased *Mbnl1* mRNA and protein expression up to ∼2-fold in C2C12 myoblasts and *HSA*^LR^ mice by stimulating *Mbnl1* promoter activity, ultimately leading to partial reversal of AS changes in several misregulated targets, correction of muscle pathology and improved muscle strength [243]. The mechanism remains unknown, but multiple predicted MEF2-binding sites within the *Mbnl1* promoter have been suggested as candidates for further analyses. Notably, vitamin D deficiency has been shown to affect the phenotype severity in DM1 patients [244], which is consistent with its putative role in the regulation of *MBNL1* expression.

Furamidine, a small molecule that binds CTG/CAG repeat DNA with nanomolar affinity, was shown to reduce the number of CUG^exp^ foci and rescue missplicing in cellular and murine models of DM1 [245]. It elevates endogenous *MBNL1* and *MBNL2* transcript and protein levels by up to 2-fold [245]. Although the mechanism has not been precisely elucidated, interactions with DNA in the *MBNL1* and *MBNL2* genes, altered TF binding or interaction directly with the *MBNL1* and *MBNL2* transcripts and their stabilization have been speculated.

### Control of MBNL protein turnover

In a DM1 *Drosophila* model, elevated MBNL levels were achieved by chloroquine (CQ)-mediated inhibition of autophagic degradation. This approach corrected AS, reduced muscle degeneration, improved muscle function, and prolonged lifespan. When added to immortalized patient-derived myoblasts, CQ increased MBNL1 and MBNL2 levels and mitigated splicing abnormalities. When applied to *HSA*^LR^ mice, CQ improved both the histological and functional phenotypes of muscles to some degree [246].

CQ was originally used as an antimalarial agent and has also proved useful in the treatment of autoimmune diseases such as systemic lupus erythematosus [247]. The price affordability, well-documented safety and tolerability profiles, and ability to ameliorate disease phenotypes in several models are advantages of repurposing this existing drug for DM1 therapy. Although hyperactivated autophagy contributes to muscle wasting and DM1 pathophysiology, patients display autophagy-related transcriptomic heterogeneity. As a result, CQ may be ineffective in certain molecular subtypes of the disease, underscoring the need for personalized therapeutic strategies [248].

### Inhibition of *MBNL*s’ translational repressors

A precision-based strategy for endogenous *MBNL* modulation was pioneered via miRNA-based tools that eliminate the direct translational repressors of *MBNL1* and *MBNL2*, namely miR-23b and miR-218, respectively [30]. Blocking their activity via complementary antisense oligonucleotides (antagomiRs or antimiRs) prevented the binding of miR-23b and miR-218 to the 3′UTRs of *MBNL1* and *MBNL2*, respectively. This, in turn, increased their protein levels, restored their cellular distribution and mitigated AS alterations in DM1 myoblasts and *HSA*^LR^ mice [30]. *In vivo* administration of antimiRs results in low toxicity, high efficacy and induced long-lasting biological effects at the molecular and functional levels [31, 32]. Recently, improved versions of antimiRs that incorporate a combination of 2′-OMe, 2′-O-methoxyethyl (MOE), and locked nucleic acid (LNA) residues throughout their sequences were additionally conjugated with fatty acids to increase their biodistribution. When tested in a cohort of primary DM1 muscle cell lines, these modified antimiRs worked therapeutically across unrelated genetic backgrounds and in cells with a wide range of repeats, thus strengthening their potential for treating multiple clinical forms of the disease [249].

Recently, ARTHEx Biotech advanced this strategy by demonstrating a dual mechanism of action of ATX-01, an antimiR that targets miR-23b. ATX-01 not only increased MBNL protein production in human DM1 myoblast cell lines and in two murine models but also reduced the levels of toxic *DMPK* mRNA. Owing to its favorable safety and biodistribution profiles in preclinical studies, ATX-01 has recently progressed to phase I/IIa clinical trials (NCT06300307) as the first antimiR-based therapeutic candidate aimed at restoring endogenous MBNL function in myotonic dystrophy.

Although antimiRs hold great potential in DM treatment, the caveat is that they may influence tumorigenesis. Therefore, their direct blocking could trigger adverse effects by affecting multiple targets apart from the intended ones [32]. Refinement of this strategy on the basis of sequence-specific targeting of miRNA binding sites within *MBNL* transcripts with so-called blockmiRs, rather than blocking miRNAs themselves via antimiRs, was recently pioneered as a safer therapeutic option [33, 250]. Such site-specific blocking was evaluated in DM1 muscle cells as well as in *HSA*^LR^ mice using blockmiRs against miR-23b and miR-218 binding sites within the 3′UTRs of *MBNL1* and *MBNL2* [33, 250]. When fused with the Pip9b2 cell-penetrating peptide for efficient delivery, blockmiRs increased *MBNL1* and *MBNL2* expression in DM1 cells and elevated protein levels in *HSA*^LR^ mice. Ultimately, this led to decreased AS abnormalities, thus reinforcing the potential of blockmiRs as DM1 therapeutics [250].

### Direct transcriptional stimulation of the *MBNL* promoter by small RNAs

An alternative approach to site-specifically enhance *MBNL1* gene expression was recently demonstrated by RNA activation (RNAa), a conserved endogenous mechanism of transcriptional gene activation [251–253] (Fig. 8). RNAa operates within the nucleus via short, promoter-directed, double-stranded RNAs termed small activating RNAs (saRNAs). The canonical RNAa pathway critically depends on Argonaute 2 protein (AGO2), which incorporates the saRNA duplex, discards the passenger (sense) strand, and forms an active complex with the guide (antisense) strand complementary to the target promoter sequence [252, 254, 255]. Upon nuclear translocation, the saRNA–AGO2 complex binds to the target site and serves as a recruitment platform for the assembly of the RNA-induced transcriptional activation (RITA) complex. RITA interacts with RNA polymerase II (RNAPII) to initiate and sustain transcriptional elongation [255] (Fig. 8).

Our recent work revealed two saRNAs, saMB1_1 and saMB1_2, which increased endogenous *MBNL1* expression in multiple cell lines, including DM1. More importantly, these saRNAs stimulated an increase in the MBNL1 protein content (up to 2–3-fold), which effectively compensated for MBNL sequestration, as evidenced by the remarkable rescue of disease-associated AS defects in DM1 patient-derived fibroblasts and myoblasts. This moderate upregulation did not cause an undesired increase in CUG^exp^ foci formation or toxicity in DM1 cells, potentially circumventing the issues associated with OE-based gene therapy tools. A comprehensive dissection of the RNAa mechanism of *MBNL1* revealed that the identified saRNA duplexes directly stimulated the transcription initiation and elongation via an on-site process involving AGO2-mediated loading of the antisense strands onto the target promoter sequences (Fig. 8). This was followed by the recruitment of RNAPII, canonical RNAa pathway components (i.e. CTR9 and RHA) and auxiliary TFs specific to the target region. Consistent with antisense strand engagement, the activity of the sense strand of the saRNA duplex as well as the lncRNA *MBNL1–AS1* overlapping *MBNL1* promoter were dispensable for RNAa of *MBNL1*. Similarly, promoter-associated cryptic transcripts in the sense orientation were apparently not involved in this process, indicating that the coding DNA strand serves as a docking site for the antisense saRNA strand and RITA complex assembly.

Putative off-target effects represent one of the major caveats associated with RNA-based therapeutics. The unexpected, AGO2-independent downregulation of the lncRNA *MBNL1–AS1*, which was partially mediated by the saRNA sense strand, could be eliminated by 5′-end biotinylation of the sense strand. This simple chemical modification inhibited the microRNA-like activity of the sense strand, reducing unintended posttranscriptional regulation while preserving saRNA therapeutic efficacy.

Notably, saRNAs targeting distinct sites located within proximity were unable to trigger *MBNL1* RNAa, likely because of the steric hindrance between two transcription complexes assembling and/or transcribing within a relatively small distance. This is contrary to RNAi technology, which often employs multiple siRNA duplexes targeting different regions of gene transcripts, often in proximity, to improve silencing efficiency. However, saRNA-mediated transcriptional stimulation was unaffected when two genes located on different chromosomes (*MBNL1* and *p21^WAF1/CIP1^*) were subjected to RNAa, demonstrating the feasibility of this technology for the parallel stimulation of *MBNL* paralogs, which in the future could significantly improve the therapeutic outcome in DM1. One additional benefit of the saRNA-based approach includes the possibility of achieving therapeutic effects with significantly lower levels of gene upregulation than those of exogenous OE, thereby reducing the likelihood of adverse effects. Taken together, these new findings warrant further studies on the RNAa of *MBNLs* and on efficient drug delivery systems for these therapeutic small RNAs.

### MBNL-centered DM therapies: criticisms and perspectives

Most of the therapies based on increasing *MBNL* expression provide valuable mechanistic insights into DM, but only a subset shows genuine clinical promise. Among these, blockmiRs and RNAa stand out for their ability to provide a balanced protein isoform repertoire as well as specificity. The efficacy and a promising safety profile of blockmiRs have already been demonstrated in preclinical mouse studies. In contrast, the lack of promoter sequence conservation between humans and mice precludes preclinical validation of *MBNL1*-directed saRNAs until suitable models become available. Encouragingly, a recent report from the Duchenne muscular dystrophy field demonstrated that systemic administration of a muscle-targeted AAV vector delivering a small activating RNA (MyoAAV-saRNA-257) significantly upregulated endogenous utrophin, a paralog of dystrophin, in a mouse model of the disease [256]. These preclinical findings establish MyoAAV-saRNA as a promising therapeutic strategy, supporting the feasibility of a similar approach to restore *MBNL1* expression in the DM1 muscle.

Compounds such as CQ, calcitriol, and furamidine show therapeutic potential but require careful evaluation of their systemic effects and unresolved mechanistic aspects in DM treatment. Nonetheless, they represent interesting examples of drug repurposing, highlighting the potential of existing compounds to provide therapeutic benefit in DM. Broad epigenetic modulators, on the other hand, remain primarily of academic interest unless their selectivity and safety are significantly improved.

The multisystemic clinical presentation of DM stands behind the concept of combinatorial therapies, which utilize two therapeutic strategies that can produce additive or even synergistic beneficial effects, alleviating the majority of disease symptoms [257]. Thus, it is tempting to speculate that coadministration of saRNAs targeting distinct MBNL paralogs or using blockmiRs/antimiRs (e.g. ATX-01) in conjunction with other drugs or therapies (e.g. focused on silencing of the toxic RNA repeats), could leverage synergistic effects in disease conditions characterized by MBNL insufficiency.

In conclusion, the DM1 field is moving towards strategies that combine mechanistic precision, moderate but functional upregulation of the MBNL, and clinically translatable delivery methods. According to the proverb, it is the dose that differentiates the poison from the remedy; therefore, strategies based on moderate or even autoregulatory MBNL enhancement, as well as efficient delivery of therapeutic compounds, should be pursued to ensure efficacy while avoiding dose-dependent adverse effects. Compounds and gene therapies with well-characterized molecular mechanisms and tolerable off-target profiles are most likely to progress successfully toward a clinical use.

## Conclusions

In this review, we summarized the current knowledge on *MBNL* genes and proteins and portrayed their key roles in a wide range of physiological and pathological conditions. These highly versatile proteins exhibit different expression patterns, tissue-specific effects, and functional diversity, underscoring the complexity of their regulatory mechanisms across cellular contexts. Accumulating evidence indicates that these essential regulators of RNA metabolism may be ample therapeutic targets in multiple pathological conditions associated with imbalance in their protein level, but with no underlying gene mutations.

In myotonic dystrophy, the most extensively studied disease associated with MBNL deficiency, precise regulation of MBNL expression is crucial and can be achieved through targeted endogenous modulation, as exemplified by the application of distinct RNA-based technologies. Such tailored augmentation may help to avoid current limitations related to damaging consequences associated with excessive MBNL activity. The future challenge lies in achieving efficient, safe and targeted delivery of such compounds, not only to muscle, but also to other tissues affected by myotonic dystrophy.

Overall, although current knowledge regarding the physiology and pathophysiology of *MBNL* genes and proteins is relatively broad, elucidating their expression regulators and developing strategies to harness their activity may foster the development of novel therapeutic targets across diverse fields, including neuromuscular diseases and cancer.

## Data Availability

No primary research results or new data were generated as part of this review.
